# Recent advances in stimuli-response mechanisms of nano-enabled controlled-release fertilizers and pesticides

**DOI:** 10.1016/j.eehl.2023.07.005

**Published:** 2023-07-23

**Authors:** Meimei Shen, Songlin Liu, Chuanjia Jiang, Tong Zhang, Wei Chen

**Affiliations:** College of Environmental Science and Engineering, Ministry of Education Key Laboratory of Pollution Processes and Environmental Criteria, Tianjin Key Laboratory of Environmental Remediation and Pollution Control, Nankai University, Tianjin 300350, China

**Keywords:** Stimuli-responsive, Controlled release, Nanocarrier, Nanofertilizer, Nanopesticide

## Abstract

Nanotechnology-enabled fertilizers and pesticides, especially those capable of releasing plant nutrients or pesticide active ingredients (AIs) in a controlled manner, can effectively enhance crop nutrition and protection while minimizing the environmental impacts of agricultural activities. Herein, we review the fundamentals and recent advances in nanofertilizers and nanopesticides with controlled-release properties, enabled by nanocarriers responsive to environmental and biological stimuli, including pH change, temperature, light, redox conditions, and the presence of enzymes. For pH-responsive nanocarriers, pH change can induce structural changes or degradation of the nanocarriers or cleave the bonding between nutrients/pesticide AIs and the nanocarriers. Similarly, temperature response typically involves structural changes in nanocarriers, and higher temperatures can accelerate the release by diffusion promoting or bond breaking. Photothermal materials enable responses to infrared light, and photolabile moieties (e.g., *o*-nitrobenzyl and azobenzene) are required for achieving ultraviolet light responses. Redox-responsive nanocarriers contain disulfide bonds or ferric iron, whereas enzyme-responsive nanocarriers typically contain the enzyme’s substrate as a building block. For fabricating nanofertilizers, pH-responsive nanocarriers have been well explored, but only a few studies have reported temperature- and enzyme-responsive nanocarriers. In comparison, there have been more reports on nanopesticides, which are responsive to a range of stimuli, including many with dual- or triple-responsiveness. Nano-enabled controlled-release fertilizers and pesticides show tremendous potential for enhancing the utilization efficiency of nutrients and pesticide AIs. However, to expand their practical applications, future research should focus on optimizing their performance under realistic conditions, lowering costs, and addressing regulatory and public concerns over environmental and safety risks.

## Highlights

• pH-responsive nanofertilizers and nanopesticides are extensively studied.
• Controlled-release nanopesticides with enzyme and redox responses are emerging.
• Various recently reported nanopesticides exhibit dual- or triple-responsiveness.
• Novel materials such as metal-organic frameworks are actively studied for preparing nanocarriers.
• Environmental risks of stimuli-responsive nanocarriers need to be evaluated.

## Graphical abstract

**Figure:**
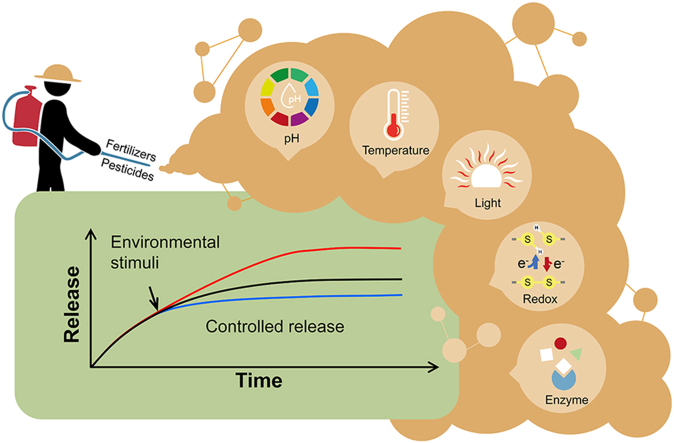

## 1 Introduction

Sustainable development of agriculture, particularly crop production, is necessary to ensure food security and nutrition for a growing world population. Over recent decades, the productivity of crop agriculture has continuously increased, owing to innovations in agricultural technologies and management [1]. However, this increase in agricultural productivity is unsustainable due to factors such as climate change [2] and the large amounts of agrochemicals (primarily chemical fertilizers and pesticides) used, which have negative impacts on ecosystems [1,3,4]. It is estimated that global agricultural production annually requires approximately 120 million tons of nitrogen fertilizers, 50 million tons of phosphate-based fertilizers, and over 2.6 million tons of pesticides [5]. The utilization efficiencies of traditional fertilizers and pesticides are typically low, e.g., 20%–50% for nitrogen, ≤25% for phosphorus, and ≤10% for pesticides [6,7]. Large portions of the nutrients from these traditional fertilizers are unavailable to crop plants due to leaching, volatilization, or interaction with soil components (e.g., soil organic matter, minerals, and enzymes/microbes) via the processes of adsorption, complexation, precipitation, and catalyzed transformation [8,9]. Likewise, the active ingredients (AIs) of traditional pesticides are prone to losses before reaching the target, through leaching, volatilization, photolysis, hydrolysis, and biodegradation [10]. The extensive use of these traditional fertilizers and pesticides has caused serious environmental problems, including the degradation of agricultural soils, the eutrophication of water bodies, a decrease in biodiversity, and the enhanced emission of greenhouse gases [1]. Therefore, it is imperative to develop fertilizers and pesticides with enhanced utilization efficiencies to maintain or even further increase the agricultural production yield while decreasing the environmental impact of agricultural practices.

The rapid development of nanotechnology has opened up new opportunities to overcome the bottlenecks in crop agriculture [1,11,12], e.g., by engineering smart plant sensors [13], enhancing the tolerance of crop plants to stress (e.g., drought [14] and salt stress [15,16]), improving soil conditions (e.g., modulating soil enzyme activity [17]), and achieving targeted delivery of agrochemicals [1]. Among these approaches, nanotechnology-enabled fertilizers and pesticides have shown great potential to effectively enhance crop nutrition and protection [[18], [19], [20], [21]]. Certain nanomaterials, such as nanoscale copper oxide or hydroxide [[22], [23], [24]], copper sulfide [25], zero-valent iron [26,27], selenium (Se) [28], manganese oxides (e.g., Mn_2_O_3_) [29], hydroxyapatite [30], and zinc oxide (ZnO) [31], can themselves serve as nanofertilizers, providing plants with micronutrients, or as nanopesticides, protecting plants from harmful bacteria, fungi, weeds, and pests [32]. In addition to the direct use of these nanomaterials as agrochemicals, there has been enormous interest in developing nano-formulations of traditional fertilizer nutrients and pesticide AIs to minimize losses and improve utilization efficiency. In particular, a variety of nanocarriers have been designed for fabricating nanofertilizers and nanopesticides with slow- or controlled-release properties, which have the potential to release plant nutrients and pesticide AIs in accordance with the needs of crops and in response to changes in environmental conditions (e.g., pH, temperature, light, and redox potential) or biological stimuli (e.g., the presence of enzymes or other biomolecules) [33]. These nanocarriers include both nanomaterials with at least one dimension in the size range of 1–100 nm (or practically up to a few hundred nanometers) and nanostructured bulk materials with nanoscale building blocks or nanopores [34]. Regarding their chemical composition, nanocarriers have been fabricated with organic (both natural and synthetic polymers), inorganic (e.g., nanoclays, mesoporous silica, and elemental carbon nanoparticles), or organic–inorganic composite materials in order to meet specific needs. Although research in agricultural nanotechnology is in its infancy, the applications of nanomaterial-based agrochemicals, particularly controlled-release nanofertilizers and nanopesticides, are anticipated to significantly decrease the environmental impact of agricultural practices and promote global eco-environmental health.

The last decade has witnessed a boom in research on controlled-release agrochemicals, and recently there have been excellent reviews on this topic detailing the preparation, applications, and prospects of controlled-release fertilizers [35] or pesticides [33,[36], [37], [38]]. However, there remains a lack of a systematic summary of the key stimuli-response mechanisms, which is indispensable for facilitating the rational design of controlled-release nanofertilizers and nanopesticides. Moreover, considering the rapid development of nanotechnology for smart agriculture, it is desirable to summarize the state-of-the-art research in this area. Herein, we present a review of the stimuli-response mechanisms and recent advances in nanofertilizers and nanopesticides with controlled-release properties. First, we systematically summarize the major categories of mechanisms underlying controlled release enabled by “smart” nanocarriers in response to different environmental and biological stimuli. Second, we summarize recent progress in the design of controlled-release nanofertilizers and nanopesticides, which are categorized according to the chemical compositions of the nanocarriers (i.e., organic, inorganic, and organic–inorganic composites), with significant advances highlighted. Finally, we discuss future research needs in the development of controlled-release nanofertilizers and nanopesticides and identify the key barriers to their practical applications.

## 2 Stimuli-response mechanisms underlying the controlled release of plant nutrients and pesticide AIs

**Fig. 1:**
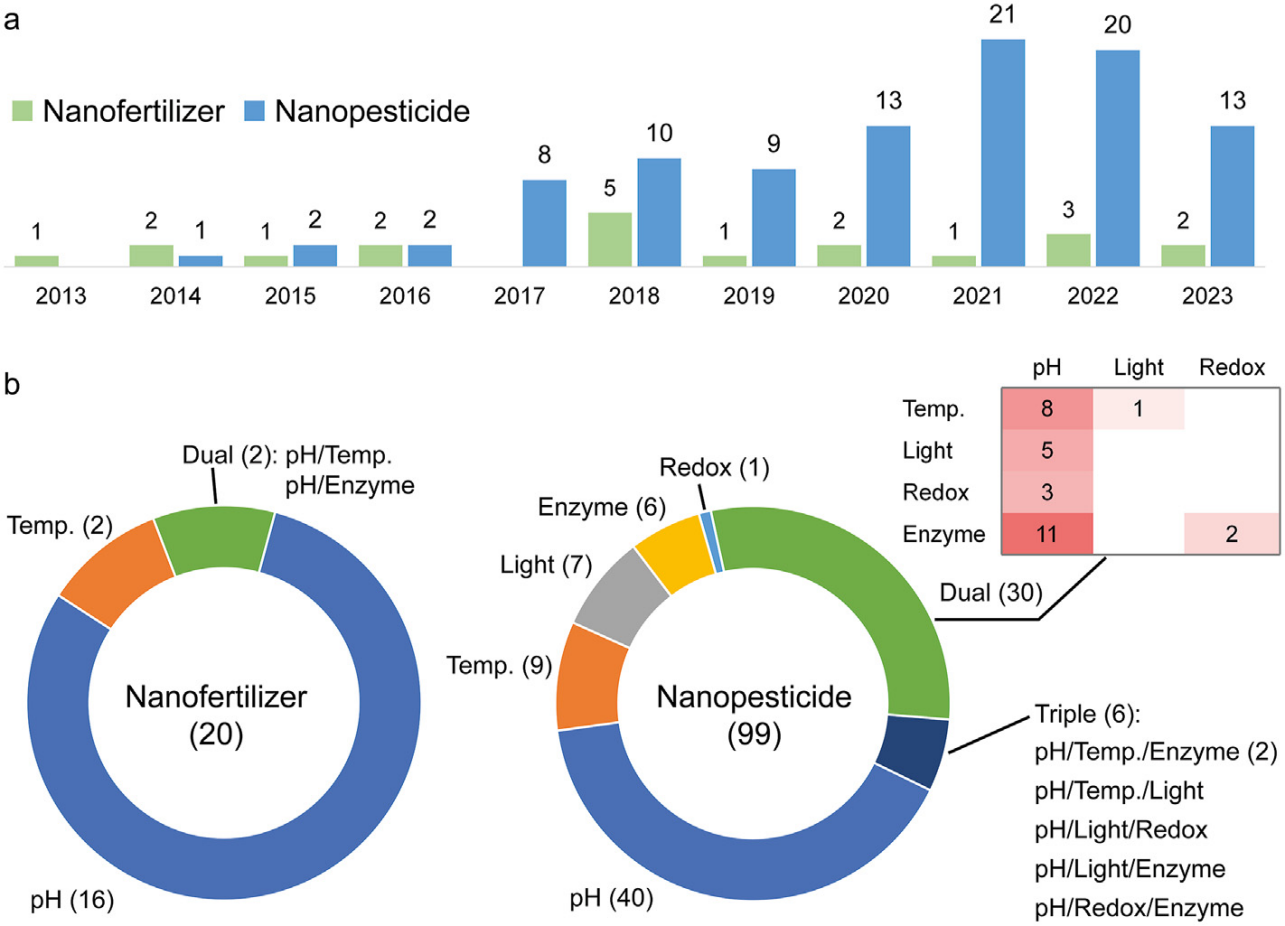
Statistics of stimuli-responsive controlled-release nanofertilizers and nanopesticides reported in the last decade, grouped by (a) the year reported and (b) the type of stimuli-responsiveness. The numbers above the columns in Part a and those in parentheses and in the heat map of Part b represent the numbers of reported nanofertilizers and nanopesticides. Temp., temperature; Dual, dual stimuli-responsive; Triple, triple stimuli-responsive. (Source: Web of Science, by June 2023).

In the past decade, a variety of nanofertilizers and nanopesticides with stimuli-responsive controlled-release properties have been developed (Fig. 1). While slow-release fertilizers, also commonly termed “controlled-release fertilizers,” have been pursued for over half a century [39,40] and are still actively studied [9,[41], [42], [43], [44]], the stimuli-responsive release of nutrients enabled by “smart” nanocarriers has only received attention in the last decade. Most studies have focused on pH-responsiveness, with only a few on temperature- or enzyme-responsiveness. Meanwhile, there has been tremendous interest in designing nanopesticides with adjustable release rates in response to various environmental or biological stimuli (e.g., pH, temperature, light, redox, and enzymes), including many with dual- or triple-response properties.

Stimuli-responsive controlled release can be achieved via two general mechanisms: (1) reversible or irreversible structural change of the nanocarrier upon external stimulation, triggering the release; and (2) the breaking of the chemical and/or hydrogen bonds between the nanocarrier and the nutrient or pesticide AI upon stimulation to release the nutrient or pesticide AI. In the following subsections, we summarize the main mechanisms underlying the responses to common environmental and biological stimuli by rationally designed nanocarriers (Table 1).

**Table 1:** The main stimuli-response mechanisms of nanocarriers for controlled release of nutrients and pesticide AIs.

Response type	Main response mechanisms	Examples of nanocarriers
pH	(1) Structural change of polymers containing carboxyl or amino groups due to (de)protonation of the groups at different pH values, which influences the hydrogen bonding and electrostatic interaction within the nanocarriers	Alginate-grafted anisotropic silica [45], alginate-*g*-poly(*N*-isopropyl acrylamide-*co*-*N*,*N*-diethylacrylamide)/semi-coke [46], alginate-chitosan [47], alginate hydrogel-dopamine-modified attapulgite [48], alginate-*g*-poly(acrylic acid-*co*-acrylamide)-clinoptilolite [49], alginate-*g*-poly(acrylic acid-*co*-acrylamide)-montmorillonite [50], aminated-cellulose nanofiber/poly(acrylamide-*co*-2-aminoethyl methacrylate) [51], banana peel cellulose-*g*-poly(acrylic acid)-polyvinyl alcohol-Mg–Al LDH [52], phosphorylated zein-carboxymethyl cellulose-*g*-poly(diallyldimethylammonium chloride) [53], carboxymethyl cellulose/3,3′-dithiobis(propionohydrazide) [54], carboxymethyl chitosan@carbon nanoparticle [55], carboxylated porous carbon nanoparticle@chitosan [56], carboxymethyl chitosan-amino-functionalized MSN [57], chitosan [58], chitosan-tripolyphosphate [59], chitosan-lignosulfonate [60], chitosan-diatomite/Fe_3_O_4_ [61], chitosan/carboxymethyl chitosan [62], chitosan-SS-zein [63], chitosan-sodium lignosulfonate@alkaline lignin-based Pickering emulsion [64], cotton stalk-*g*-poly(acrylic acid)-polyvinylpyrrolidone-bentonite [65], 3,4-dihydroxyhydrocinnamic acid, *N*-hydroxysuccinimide ester modified chitosan [66], GO-carboxymethyl chitosan [67], GO@polydopamine [68], HMS-SS-chitosan oligosaccharide [69], HMS@1-tetradecanol@polydopamine [70], isolated soy protein/carboxymethyl chitosan [71], lignosulfonate-chitosan-polydopamine [72], lignosulfonate/dodecyl dimethyl benzyl ammonium chloride/Fe(III) [73], maize bran-*g*-poly(acrylic acid-*co*-acrylamide)-montmorillonite [74], mesoporous nano-selenium@polyacrylic acid [75], MoS_2_@MSN@cyclodextrin polymer [76], *O*-carboxymethyl chitosan [77], polydopamine-poly(*N*,*N*-dimethylaminoethyl methacrylate) [78], porous porphyrinic MOFs@pectin@chitosan [79], poly(acrylic acid)-*b-*poly(*N*-isopropyl acrylamide) [80], poly[2-(2-bromoisobutyryloxy)-ethyl methacrylate-*g*-poly(acrylic acid)-*b*-poly(*N*-isopropyl acrylamide)] [81], poly(*β*-cyclodextrin)-adamantane-grafted poly(acrylic acid) [82], poly(glycidyl methacrylate-*co*-acrylic acid)-HMS [83], polyethyleneimine-grafted lignin [84], polyacrylamide/methylcellulose/calcic montmorillonite [85], polyethylenimine modified hollow/mesoporous carbon nanoparticle [86], polydopamine-attapulgite-calcium alginate [87], rGO-Cu_2–*x*_Se@chitosan [88], sulfonated-carboxymethyl cellulose-*g*-poly(acrylic acid)-polyvinylpyrrolidone-silica nanoparticle [89], salep-*g*-poly(acrylic acid)/montmorillonite [90], subabul stem lignin [91], UiO-66-NH_2_-carboxymethyl cellulose [92]

	(2) Degradation of the nanocarriers due to acid- or base-mediated decomposition of their components	Alginate-Mg–Al LDH [93], bovine serum albumin nanoparticle [94], carboxymethyl chitosan-allyl glycidyl ether-trisiloxane surfactant [95], α-cyclodextrin-HMS [96], α-cyclodextrin/ZIF-8 [97], 2,4-dinitrobenzaldehyde@ZIF-8 [98], Fe-doped MSN/tannic acid [99], γ-FeOOH@biochar [100], glycine methyl ester-conjugated polysuccinimide nanoparticle [101], halloysite nanotube/Ca^2+^/EDTA^2−^/calcium alginate [102], 3-mercaptopropyl trimethoxysilane and poly(ethylene glycol) diacrylate functionalized boron nitride nanoplatelet [103], methoxypolyethylene glycol-*o*-nitrobenzyl [104], Mg–Al LDH [105], MSN-polydopamine-Cu^2+^ [106], MSN/β-glucan [107], MIL-101(Fe)@silica [108], MIL-101(Fe)-carboxymethyl starch [109], MIL-101(Fe)-polydopamine [110], MIL-101(Fe)-tannic acid [111], MIL-101@carboxymethyl chitosan [112], polydopamine@NH_2_-MIL-101(Fe) [113], soybean protein isolate-carboxymethyl cellulose [114], trimethylammoniumpillar[5],arene-methyl orange-functionalized mesoporous selenium [115], UiO-66@hydroxypropyl cellulose [116], ZIF-8 [[117], [118], [119]], ZnO@ZIF-8 [120]

	(3) pH-induced cleavage of the chemical or hydrogen bonds linking the nutrients or pesticide AIs and the nanocarriers	Aldehyde-functionalized ZnO quantum dot [121], carboxyl cellulose-attapulgite [122], polydopamine-isocyanatopropyltriethoxysilane-polyethyleneimine [123], sulfonate-functionalized MSN [124], trimethylammonium-functionalized MSN [125]

Temperature	(1) Structural change of nanocarriers composed of temperature-sensitive polymers with an LCST or of liposomes with a suitable phase transition temperature	Alginate-*g*-poly(*N*-isopropyl acrylamide-*co*-*N*,*N*-diethylacrylamide)/semi-coke [46], ASO-ethylene oxide/propylene oxide block copolymer-ferroferric oxide-palygorskite [126], carboxymethyl cellulose/poly(*N*-vinylcaprolactam-*co*-acrylamide) [127], isopropyl myristate@poly(*N*-isopropylacrylamide-*co*-butyl methylacrylate) [128], MSN@poly(*N-*isopropyl acrylamide) [129] poly(acrylic acid)-*b-*poly(*N*-isopropyl acrylamide) [80], poly[2-(2-bromoisobutyryloxy)-ethyl methacrylate-*g*-poly(acrylic acid)-*b*-poly(*N*-isopropyl acrylamide)] [81], poly(*β*-cyclodextrin)-adamantane-grafted poly(acrylic acid) [82], polydopamine-poly(*N*-isopropyl acrylamide) [10], polydopamine-poly(*N*,*N*-dimethylaminoethyl methacrylate) [78], polyether polyol/polycaprolactone [130], poly(*N*-isopropyl acrylamide-*co*-methacrylic acid)-HMS [131], poly(*N*-isopropyl acrylamide)-GO [132], poly(propylene oxide-*co*-carbon dioxide-*co*-allyl glycidyl ether)-poly(*N*-isopropylacrylamide)-polyethylene glycol monomethyl ether [133], yolk lecithin-cholesterol [134]
		
	(2) Accelerated diffusion of nutrients or pesticide AIs within the nanocarrier at higher temperatures	Calcium alginate [135], chitosan [58,75], chitosan-gum Arabic [136], n-hexadecane/nanofibrillated cellulose [137], poly(vinyl alcohol)-ASO-attapulgite [138], trimethylammonium-functionalized MSN [125]
		
	(3) Bond breaking between a pesticide AI and the nanocarrier at higher temperatures	Aldehyde-functionalized ZnO quantum dot [121], carboxylated porous carbon nanoparticle@chitosan [56]
		
Light	(1) Near-infrared-induced temperature increase of a photothermal material and subsequent temperature response	Alginate-*g*-poly(*N*-isopropyl acrylamide-*co*-*N*,*N*-diethylacrylamide)/semi-coke [46], biochar@soybean oil-polysulfide [139], Cu_2-*x*_Se-rGO [140], GO@polydopamine [68], HMS@1-tetradecanol@polydopamine [70], hollow carbon microsphere@polyethylene glycol/α-cyclodextrin [141], MIL-101(Fe)-tannic acid [111], MoS_2_@MSN@cyclodextrin polymer [76], polydopamine-poly(*N*-isopropyl acrylamide) [10], polydopamine-Ti_3_C_2_T_*x*_ [142]
		
	(2) Structural change of polymers containing photolabile moieties under UV irradiation	Biochar-azobenzene-ASO-attapulgite [143], cucurbit[8]uril/azobenzene derivative [144], 2,4-dinitrobenzaldehyde@ZIF-8 [98], methoxypolyethylene glycol-*o*-nitrobenzyl [104], 2-nitrobenzyl succinate-carboxymethyl chitosan [145]
		
	(3) Light-induced bond breaking between a pesticide AI and the nanocarrier	Poly(ethylene glycol)-*ο*-nitrobenzyl [146], thioacetal *o*-nitrobenzaldehyde [147]
		
Redox	Structural change of nanocarriers containing redox moieties [e.g., disulfide bonds, Fe(III)]	Carboxymethyl cellulose/3,3*′*-dithiobis(propionohydrazide) [54], chitosan-SS-zein [63], disulfide bond-linked and starch-coated MSN [148], HMS-didecyl disulfide [149], HMS-SS-chitosan oligosaccharide [69], iron(III)-based MOFs-pectin [150], MIL-101(Fe)-carboxymethyl starch [109], MIL-101(Fe)-tannic acid [111]
		
Enzyme	Enzyme-catalyzed degradation of nanocarriers containing enzyme substrates	Cellulose acetate/chitosan/zein/starch/polycaprolactone [151], chitosan-lignosulfonate [60], chitosan-sodium lignosulfonate@alkaline lignin-based Pickering emulsion [64], α-cyclodextrin-HMS [96], α-cyclodextrin/ZIF-8 [97], α-cyclodextrin-phenylamine-functionalized HMS [152], disulfide bond-linked and starch-coated MSN [148], HMS-hydroxypropyl cellulose [153], iron(III)-based MOFs-pectin [150], lignin [154], lignin/polysaccharide/Fe(III) [155], lignosulfonate/dodecyl dimethyl benzyl ammonium chloride/Fe(III) [73], MIL-101(Fe)-carboxymethyl starch [109], MoS_2_@MSN@cyclodextrin polymer [76], MSN-chitosan [156], MSN/β-glucan [107], MSN-pectin [157], *N*-succinyl chitosan [158], poly(*β*-cyclodextrin)-adamantane-grafted poly(acrylic acid) [82], polydopamine-isocyanatopropyl triethoxysilane-polyethyleneimine [123], porous porphyrinic MOFs@pectin@chitosan [79], UiO-66@hydroxypropyl cellulose [116], zein nanocapsule [159], zein nanoparticle [160]

AI, active ingredient; ASO, amino silicon oil; HMS, hollow mesoporous silica; LCST, lower critical solution temperature; LDH, layered double hydroxides; MIL-101, Materials Institute Lavoisier-101; MOFs, metal-organic frameworks; MSN, mesoporous silica nanoparticle; (r)GO, (reduced) graphene oxide; UiO-66, University of Oslo-66; UV, ultraviolet; ZIF-8, zeolitic imidazolate framework-8.

### 2.1 pH response

Soil pH affects a range of abiotic and biotic processes in croplands, thus influencing the fate and efficacy of nutrients and pesticide AIs. For example, pH has a significant influence on the volatilization of ammonia formed from urea applied to agricultural soil [161], and an ammonia- or urea-based fertilizer capable of slow release at a relatively high pH (e.g., pH > 7.0) is expected to reduce nitrogen loss due to volatilization. Pesticide AI release triggered by a specific pH condition corresponding to the digestive organs of pests [162] or infected crop plants [75,120] can improve the efficacy of pesticides. Thus, it is desirable to design fertilizers and pesticides with pH-response properties, from which the release of nutrients and pesticide AIs can be triggered by a change in pH.

The most common types of pH-responsive (nano)fertilizers and (nano)pesticides consist of polymers containing acidic (e.g., carboxyl) or basic (e.g., amino or, less commonly, pyridyl) [163] functional groups with acid dissociation constants (i.e., p*K*_a_) falling within environmentally or biologically relevant pH ranges. Various natural (e.g., alginate), synthetic [e.g., poly(acrylic acid) (PAA) and poly(methacrylic acid) (PMAA)], and modified natural polymers (e.g., carboxymethyl cellulose) contain carboxyl groups. At a pH lower than its p*K*_a_, the carboxyl group is predominantly protonated (i.e., as –COOH), and hydrogen bonds form between –COOH groups or between –COOH and other groups (e.g., hydroxyl groups) in the polymer, hindering the release of loaded nutrients or pesticide AI. At a pH higher than the p*K*_a_, the carboxyl group is predominantly deprotonated (i.e., as –COO^–^), and electrostatic repulsion between the –COO^–^ groups changes the conformation of the polymer (observed as “swelling”), providing pore channels for accelerated release. However, this acceleration effect may be partially counteracted by a longer diffusion pathway in a “swollen” nanocarrier. It is noted that the presence of cations (especially multivalent cations such as Ca^2+^ and Fe^3+^) can screen the electrostatic repulsion between the –COO^–^ groups, thus inhibiting the “swelling” of the polymer and attenuating the pH response [74]. Other natural or synthetic polymers, such as chitosan, poly(*N*,*N*-dimethylaminoethyl methacrylate) (PDMAEMA), and poly(*N*,*N*-diethyl aminoethyl methacrylate), contain abundant amino groups [59,61]. Nanocarriers based on these polymers swell at lower pH due to electrostatic repulsion between amino groups that are predominantly protonated (i.e., as –NH_3_^+^), leading to a higher release rate of loaded nutrients or pesticide AIs. For polymers containing both carboxyl and amino groups (e.g., carboxymethyl chitosan), the pH response also follows the above mechanisms, but the overall effect may be controlled by the (de)protonation of one type of functional group (e.g., carboxyl group) in a specific pH range [57].

The pH-responsive controlled release can also be achieved using nanocarriers consisting of materials prone to acid- or base-catalyzed decomposition (e.g., via hydrolysis or dissolution) [103,164]. For example, some organic materials containing ester [103], Si–O–Si [95], or metal-N [117] bonds can undergo extensive hydrolysis under acidic or basic conditions, whereas some minerals, such as Mg–Al layered double hydroxides (LDH), can partially dissolve at lower pH, resulting in faster release of loaded nutrients [93,105].

In addition to pH-induced structural changes of nanocarriers, pH-controlled release can be realized by cleavage of the chemical or hydrogen bonds linking the nutrients or pesticide AIs and nanocarriers with acidic or basic functional groups. For example, in an oxidized cellulose-loaded ferrous iron [Fe(II)] fertilizer, the Fe(II) ions are chelated by carboxyl groups, and the chelation is weakened at lower pH (e.g., pH 4–5), resulting in enhanced release of Fe(II) [122]. In addition, the surface microstructure [122] and hydrophobicity [75,78] of nanocarriers can change with pH, which may enable pH-dependent adhesion properties of (nano)fertilizers and (nano)pesticides, further increasing their utilization efficiency.

### 2.2 Temperature response

Developing temperature-responsive nanofertilizers and nanopesticides is desirable because temperature can have both direct and indirect effects on the efficiency of fertilizers and pesticides. For example, it is expected that losses of volatile nutrients and pesticide AIs are accelerated at higher temperatures (e.g., during the daytime), and nanofertilizers and nanopesticides that exhibit faster release at lower temperatures may thus reduce their losses and enhance their utilization efficiency.

**Fig. 2:**
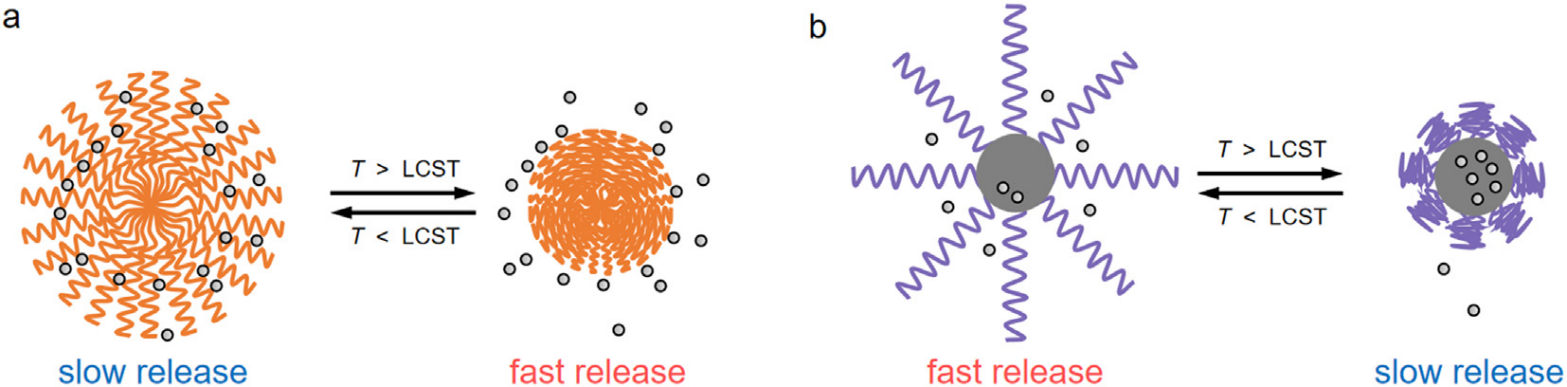
Schematic diagram of temperature-responsive controlled release of nutrients or pesticides AIs enabled by nanocarriers consisting of temperature-sensitive polymers with a lower critical solution temperature (LCST). (a) In this case, the nutrient or pesticide AI is loaded into the network of the polymeric nanocarrier. At a temperature below the LCST, the swollen network hinders the release of nutrient/pesticide AIs; at a temperature above the LCST, the polymer chains collapse, resulting in faster release. (b) In this case, the nutrient or pesticide AI is encapsulated by the temperature-sensitive polymer coating. At a temperature above the LCST, the compactly coated polymers hinder the release of the nutrient/pesticide AI, whereas at a temperature below the LCST, the unfolded polymer chains allow faster release.

The temperature response is primarily achieved by incorporating temperature-sensitive polymers into the carrier, such as poly(*N*-isopropyl acrylamide) (PNIPAm) [10,80,129,131,132], ethylene oxide/propylene oxide block copolymer (F-127) [126], and PDMAEMA [78]. A temperature-sensitive polymer has one (or, in some cases, more than one) [165,166] critical solution temperature at which the polymer undergoes a phase transition, leading to a drastic change in its miscibility/solubility in a solvent (e.g., water) [78,163]. Temperature-sensitive polymers may have a lower critical solution temperature (LCST), below which they become miscible/soluble in a solvent, or an upper critical solution temperature (UCST), above which they become miscible/soluble [163]. Typically, temperature-sensitive polymers with an LCST are used for fabricating temperature-responsive nanocarriers, which shrink or collapse at a temperature above the LCST, rendering either enhancement [10,80,131] (Fig. 2a) or inhibition [78] (Fig. 2b) of release with increasing temperature. Temperature-sensitive polymers with a UCST or with both an LCST and a UCST have rarely been explored for fabricating temperature-responsive nanofertilizers and nanopesticides, though their unique temperature-response behaviors may be useful in some scenarios. Although the critical solution temperature(s) of a polymer depends on the type and properties of the matrix (e.g., alcohol versus water and the pH of an aqueous solution) [78,165,166], it is feasible to rationally design polymers with desired critical solution temperature(s) and correspondingly tailored temperature-response properties by controlling the type and content of temperature-sensitive monomers during the copolymerization process [163,167]. Similarly, liposome nanovesicles composed of lipids with a suitable phase transition temperature can also exhibit temperature-response behavior [134].

Temperature-dependent release has also been observed for nanocarriers made of other types of materials, since higher temperatures can typically increase the diffusivity of a nutrient or pesticide AI within a nanocarrier and in the aqueous medium. For example, even for polymers without a critical solution temperature, such as chitosan/gum Arabic composite nanoparticles [136], a higher temperature can induce relaxation of the polymer chains and increase the diffusivity of the loaded chemicals. In addition, temperature-dependent controlled release can be achieved through temperature-induced bond breaking and consequent uncoupling of the nutrient or pesticide AI from the nanocarrier [56,121].

### 2.3 Light response

Light can affect the behaviors (e.g., feeding) of insects [168], and thus light-responsive controlled-release nanopesticides can enhance the utilization efficiency of pesticide AIs. Nanopesticides capable of faster release under light irradiation are expected to be more effective in controlling diurnal pests, whereas those exhibiting faster release in the dark may control nocturnal pests more efficiently. Light-responsive (or photoresponsive) controlled release can be achieved by constructing nanocarriers with (1) photothermal materials that induce a temperature increase under light irradiation, such as polydopamine (PDA) [10,68,70,142], graphene oxide (GO) and other carbon-based nanomaterials [68,140,141], and certain metal-based semiconductors (e.g., Cu_2-*x*_Se, MoS_2_, and Ti_3_C_2_T_*x*_) [76,140,142] or (2) polymers containing photolabile moieties (e.g., *o*-nitrobenzyl group [98,104,[145], [146], [147]] and azobenzene [143,144]) that undergo photolysis or conformational change under light irradiation. For the first type, a notable example is the PDA-PNIPAm nanocomposite, which exhibits near-infrared (NIR) and temperature dual response [10]. For the second type, the photolabile moiety can either be part of a core-shell nanocarrier to encapsulate the pesticide AI [98,104,[143], [144], [145]] or serve as a linker to conjugate the pesticide AI with the nanocarrier [146,147]. For instance, the *o*-nitrobenzyl group can undergo photolysis under ultraviolet (UV) irradiation, changing the structure of the nanocarrier [145] or breaking the linkage between the polymeric nanocarrier and the pesticide AI [146,147]. Azobenzene can undergo *trans*-*cis* and *cis*-*trans* isomerization simultaneously under UV-visible light irradiation, serving as light-motivated “stirrers” to promote the release of loaded pesticide AIs [143]. Note that some pesticide AIs are vulnerable to photolysis, and nanocarriers that can prevent their decomposition under UV irradiation are desired [103,112,169]. Light-responsive fertilizers have rarely been reported. In a ferric iron-polysaccharide hydrogel-loaded phosphate fertilizer, the photochemical reaction between ferric iron and the carboxyl group can lead to degradation of the hydrogel and the light-responsive release of phosphate [170].

### 2.4 Redox and enzyme responses

The presence of a particular redox condition or an enzyme in an organism or the rhizosphere of plants can be utilized in the development of pesticides or fertilizers with targeted delivery to specific pests, weeds, or crops. In recent years, there has been increasing interest in developing redox- and enzyme-responsive controlled-release nanoagrochemicals (primarily nanopesticides) (Fig. 1 and Table 1).

The redox-responsive nanocarriers typically contain disulfide bonds (-SS-) and can respond to reducing conditions [54,63,69,148,149], as indicated by elevated concentrations of reduced glutathione in an organism [148]. Alternatively, nanocarriers composed of Fe(III)-based metal-organic frameworks (MOFs), such as Materials Institute Lavoisier-101 (MIL-101), undergo reductive decomposition at high reduced glutathione concentrations [109,150] or degradation via Fenton-like reactions in the presence of hydrogen peroxide [111], enabling redox-responsive controlled release of pesticide AIs.

Nanocarriers that can respond to a certain enzyme typically contain the enzyme’s substrate as a building block. For example, pesticides loaded in (hollow) mesoporous silica nanoparticles (MSNs) modified with biodegradable carbohydrates (e.g., α-cyclodextrin [76,96,152], starch [148], β-glucan [107], functionalized cellulose [153], or chitosan [156]) as “gatekeepers” can exhibit controlled release upon exposure to enzymes such as α-amylase [76,96,148,152], β-glucanase [107], cellulase [153], or esterase [156], due to enzymatic hydrolysis of these carbohydrates/polysaccharides. Similarly, nanocarriers composed of zein proteins can be decomposed by enzymes (e.g., trypsin) in the gut of certain insects, thus enabling the controlled release of AIs upon ingestion by these insects [160]. Most recently, MOFs modified with biomolecules have also been extensively investigated as nanocarriers for enzyme-responsive controlled-release pesticides [79,97,109,116,150].

## 3 Recent advances in stimuli-responsive controlled-release nanofertilizers

Due to advances in nanotechnology, various organic and organic–inorganic composite nanocarriers with stimuli-response properties (primarily pH-responsive) have been explored for preparing controlled-release fertilizers (Tables S1 and S2), whereas few inorganic nanocarriers have been reported [105], primarily due to the structural rigidity of inorganic materials, which is generally unfavorable for achieving stimuli-responsiveness. A majority of the studies have focused on macronutrients, such as ammonium, urea, phosphate, and nitrogen-phosphorus-potassium (NPK) compound fertilizer, and there is a smaller but growing number of studies on micronutrients, such as boron (B) [93], copper (Cu) [151], iron (Fe) [122,126], zinc (Zn) [72,78], and Se [86], as well as on plant biostimulants [118]. In the following subsections, we highlight significant advances in stimuli-responsive controlled-release fertilizers with different types of nanocarriers (i.e., organic and organic–inorganic composite nanocarriers).

### 3.1 Controlled-release fertilizers with organic nanocarriers

Organic nanocarriers for controlled-release fertilizers are copolymers or composites of synthetic polymers (e.g., PDMAEMA [78] and polycaprolactone), natural polymers (e.g., chitosan [72], PDA [72,78], zein, and starch [151]), and modified natural polymers (e.g., lignosulfonate [72] and cellulose acetate [151]). Recently, MOFs, such as zeolitic imidazolate framework-8 (ZIF-8), have also been explored as nanocarriers (Table S1) [117,118].

All the polymeric nanocarriers exhibit pH-response characteristics, except for a recently reported nanostructured hydrogel composed of carboxymethyl cellulose/poly(*N*-vinylcaprolactam-*co*-acrylamide) that exhibited temperature-responsive release of urea [127]. Notably, a pH and temperature dual-responsive nanofertilizer was prepared by grafting PDMAEMA onto PDA-coated zinc ammonium phosphate via atom transfer radical polymerization [78]. At a given temperature (i.e., 25 or 40 °C), the nutrient release rate increased with lower pH due to a reduced hindrance to mass transfer by the stretched PDMAEMA chains with an amino group (following mechanisms described in *Section 2.1*). Moreover, the nanofertilizer exhibited pH-dependent temperature responses. Under alkaline conditions (at pH 10), the nutrient release rates were accelerated at a lower temperature (25 °C) compared to a higher temperature (40 °C). Under acidic and neutral conditions (at pH 4 and 7), the release rates were slightly higher at 40 °C than at 25 °C. This difference can be explained by the fact that the LCST of PDMAEMA is pH-dependent, which is 70, 55, and 35 °C at pH 3, 7, and 10, respectively. Thus, only at pH 10 does the pH response of the nanocarrier follow the mechanisms illustrated in Fig. 2b. This example shows that there is an opportunity to construct “smart” nanofertilizers that can adapt to complex environmental conditions by utilizing multi-responsive composite materials as the nanocarrier.

Another notable example is a biopolymer-based core–shell-structured nanocarrier with pH and enzyme dual-responsiveness for the controlled release of both macronutrients (NPK) and micronutrients (Cu). Pot experiments demonstrated that the controlled-release nanofertilizer enhanced the photosynthesis of two model crop plants (i.e., soybean and wheat) compared to conventional ionic fertilizers applied at much higher concentrations [151]. Moreover, the nutrient release kinetics can be fine-tuned by changing the polymer composition and distribution of the nutrients within the nanostructures, thereby exhibiting potential for meeting the needs of different crop species.

### 3.2 Controlled-release fertilizers with organic–inorganic composite nanocarriers

Compared with the use of organic material alone as the nanocarrier, it is more common to use organic–inorganic composites for preparing controlled-release fertilizers (Table S2). The main organic components used are alginate [49,50,93], cellulose derivatives [52,65,74,85,90,122], and PAA [52,65,74,90] and its derivatives (e.g., poly(methacrylic acid) and polyacrylamide [85]), whereas the most frequently used inorganic components are nanoclays and Mg-Al LDH. The incorporation of natural polymers (e.g., alginate, salep, and cellulose) as an ingredient is a common practice due to their environmental compatibility and safety, especially considering that the nanocarriers may be translocated or even directly applied to edible parts of crop plants. The incorporation of inorganic components not only lowers the cost of nanofertilizers [49,50,90], but can also increase the loading capacity of nutrients, owing to the porous structure and large specific surface area of nanoclay materials such as palygorskite [126] and montmorillonite [85]. Moreover, the introduction of inorganic materials can improve the thermal stability [126,171], mechanical strength [52,85], and durability [172] of the composite nanocarriers. Most composite nanocarriers are hydrogels, which have good prospects for application in arid areas because they not only have controlled-release capabilities but can also improve the water retention of soil.

**Fig. 3:**
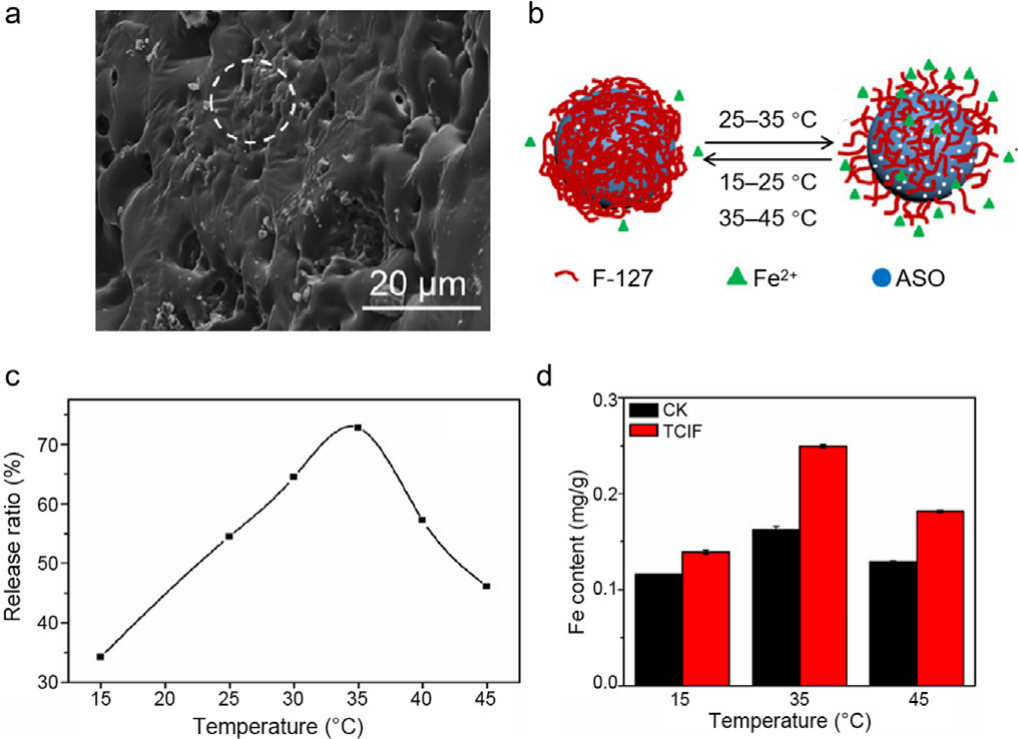
(a) Scanning electron microscopy image of temperature-responsive controlled-release iron fertilizer (TCIF). (b) Temperature-response mechanism of TCIF enabled by temperature-sensitive polymer F-127 grafted on the surface of porous amino silicon oil (ASO) shell. The TCIF exhibited (c) temperature-dependent controlled release of Fe(II) and led to (d) increased Fe content in maize shoots as compared to control (CK) in a temperature-dependent manner. Panels a–d adapted with permission from ref. [126]. Copyright (2018) Elsevier.

A majority of the organic–inorganic composite nanocarriers exhibit pH responsiveness due to their abundant acid/base functional groups in the organic component and/or the pH-sensitive structure of the inorganic component, whereas only a few composite nanocarriers exhibit temperature- [126] or anion-response [86] properties. Notably, a composite nanocarrier comprised of a palygorskite/Fe_3_O_4_ core and a porous shell of amino silicon oil (ASO)/F-127 enabled the temperature-dependent controlled release of Fe(II) [126]. As shown in Fig. 3, at temperatures below 25 °C or above 35 °C, the F-127 chains collapse and cover a majority of the pores in the ASO shell, thus inhibiting Fe(II) release. At temperatures between 25 and 35 °C, the polymer chains of F-127 stretch, uncovering the pores and allowing for the fast release of Fe(II). Moreover, pot experiments demonstrated that the controlled-release nanofertilizer promoted Fe(II) uptake and the growth of maize in a temperature-dependent manner.

## 4 Recent advances in stimuli-responsive controlled-release nanopesticides

Compared to nanofertilizers, there have been a considerably larger number of studies on stimuli-responsive controlled-release nanopesticides (Fig. 1 and Tables S3–S5). Moreover, organic materials are more frequently used alone as nanocarriers, possibly because most pesticide AIs are organic compounds that can be more easily loaded into organic carriers in a controlled manner and at a higher loading efficiency. Most of these studies have focused on synthetic pesticides as the AIs, but there are also explorations of plant-derived pesticides, e.g., limonene and carvacrol [160], eugenol [173], eupatorium adenophorum extract [174], and rotenone [175].

### 4.1 Controlled-release pesticides with organic nanocarriers

Organic nanocarriers for controlled-release pesticides are primarily composed of polymeric materials with good biocompatibility and/or biodegradability, such as PDA [10,123], alginate [47,135], chitosan [59,75,112,145] and its derivatives [95], zein [53,63,160], polysaccharides (e.g., gum Arabic) [136], and bovine serum albumin [94] (Table S3). In the last few years, MOFs [including MIL-101(Fe), University of Oslo-66 (UiO-66), and ZIF-8] have also been explored for fabricating stimuli-responsive nanocarriers. Unlike polymeric nanocarriers for nanofertilizers, which are often prepared in the form of hydrogels, those for nanopesticides are mostly in the form of core–shell structures, nanocapsules, nanomicelles, and nanoethosomes. These core–shell-structured nanocarriers can effectively inhibit the degradation of pesticide AIs (e.g., photolysis under UV irradiation) [53]. Moreover, nanocapsules or nanomicelles composed of amphiphilic polymers are especially suitable for the encapsulation of lipophilic pesticides, achieving both high loading and good dispersity in aqueous media [95].

The use of organic materials containing carboxyl or amino groups as nanocarriers can achieve the pH-responsive release of pesticide AIs. For example, a pH-responsive nanoformulation of imidacloprid was prepared by encapsulating this insecticide into poly(citric acid)-poly(ethylene glycol)-poly(citric acid) triblock linear dendritic copolymers, which exhibited a higher release rate at pH 10 than at pH 7 [162]. This nanopesticide demonstrated superior efficacy in controlling the lesser mulberry pyralid (*Glyphodes pyloalis*), primarily because the alkaline environment (pH 10) in the gut of *G. pyloalis* can trigger controlled release and thus increase the utilization efficiency of imidacloprid. As another notable example, accelerated release of avermectin encapsulated in trisiloxane-carboxymethyl chitosan nanocomposites was observed at pH 5 and 9 as compared to pH 7 due to the acid- or base-catalyzed cleavage of the Si–O–Si bond and consequential disintegration of the nanocomposite [95].

**Fig. 4:**
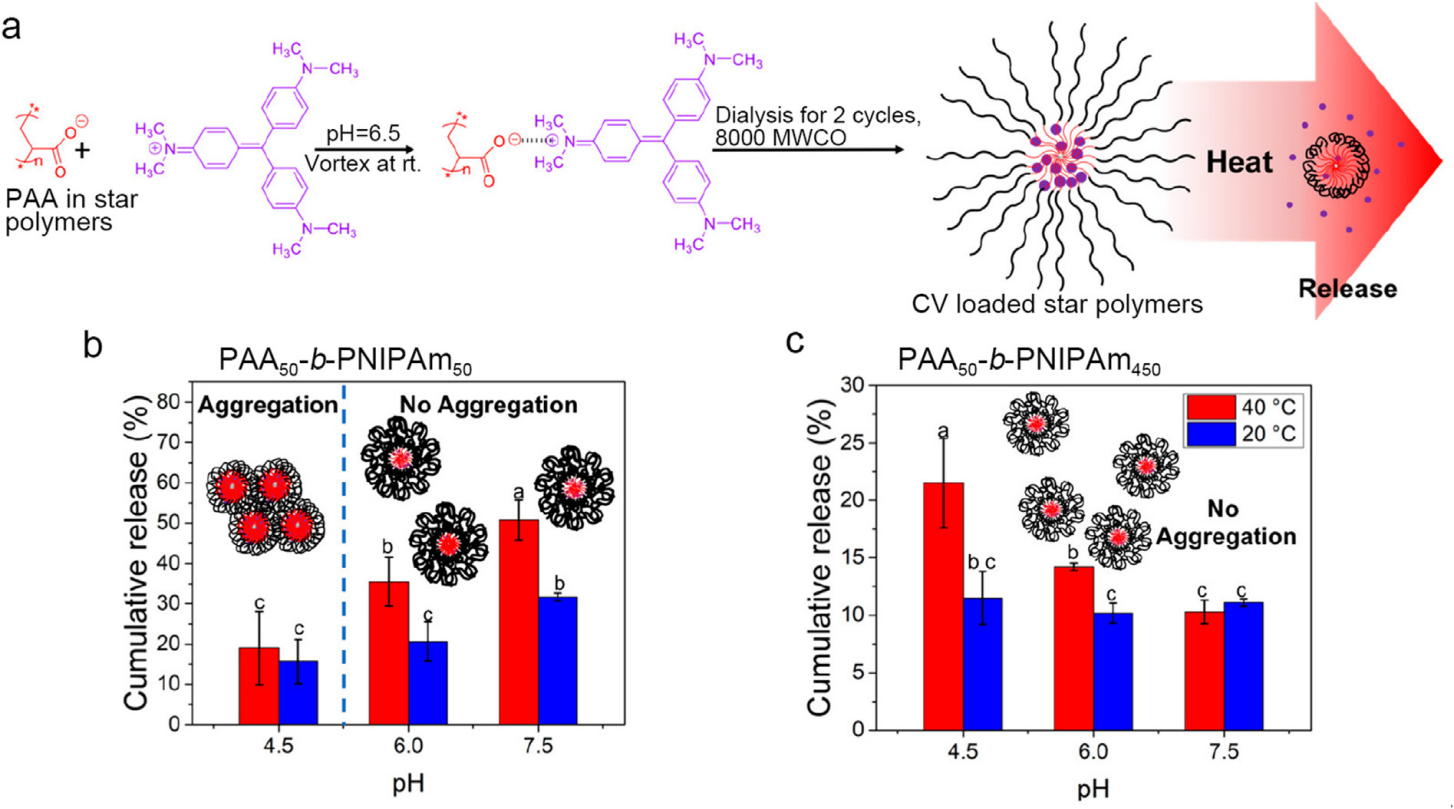
(a) Schematic diagram of the crystal violet (CV) loading process and release mechanism of PAA-*b*-PNIPAm copolymers, and the cumulative release of CV in copolymers with different PNIPAm chain lengths, i.e., (b) PAA_50_-*b*-PNIPAm_50_ and (c) PAA_50_-*b*-PNIPAm_450,_ at different pH (4.5, 6.0, and 7.5) and temperature (20 and 40 °C). Panels a–c are reprinted in part with permission from ref. [80]. Copyright (2020) American Chemical Society. PAA-*b*-PNIPAm, poly(acrylic acid)-block-poly(*N*-isopropyl acrylamide).

In addition, nanopesticides with other types of responses can be obtained using organic carriers. Recently, a temperature- and pH-responsive nanocarrier was developed using PAA-*b*-PNIPAm copolymers with different block length ratios, allowing the release of pesticide AIs to plant compartments with different pH values and temperatures (Fig. 4) [80]. The controlled release behavior of crystal violet (CV), a model antimicrobial agent, from the nanocarrier depended not only on pH (4.5, 6.0, and 7.5) and temperature (20 and 40 °C) but also on the chain composition. Under the same temperature and pH conditions, the longer the chain of PNIPAm, the slower the release rate of CV. It is worth noting that the nanocarriers have shown good foliar uptake and translocation abilities in tomato plants, indicating good application potential in the treatment of plant root diseases with foliar-applied pesticides. In addition, this synthesis strategy has been utilized to prepare bottlebrush-like polymeric nanocarriers, enabling the temperature-responsive release of spermidine, a stress-regulating agent that protects crop plants from heat stress [81].

**Fig. 5:**
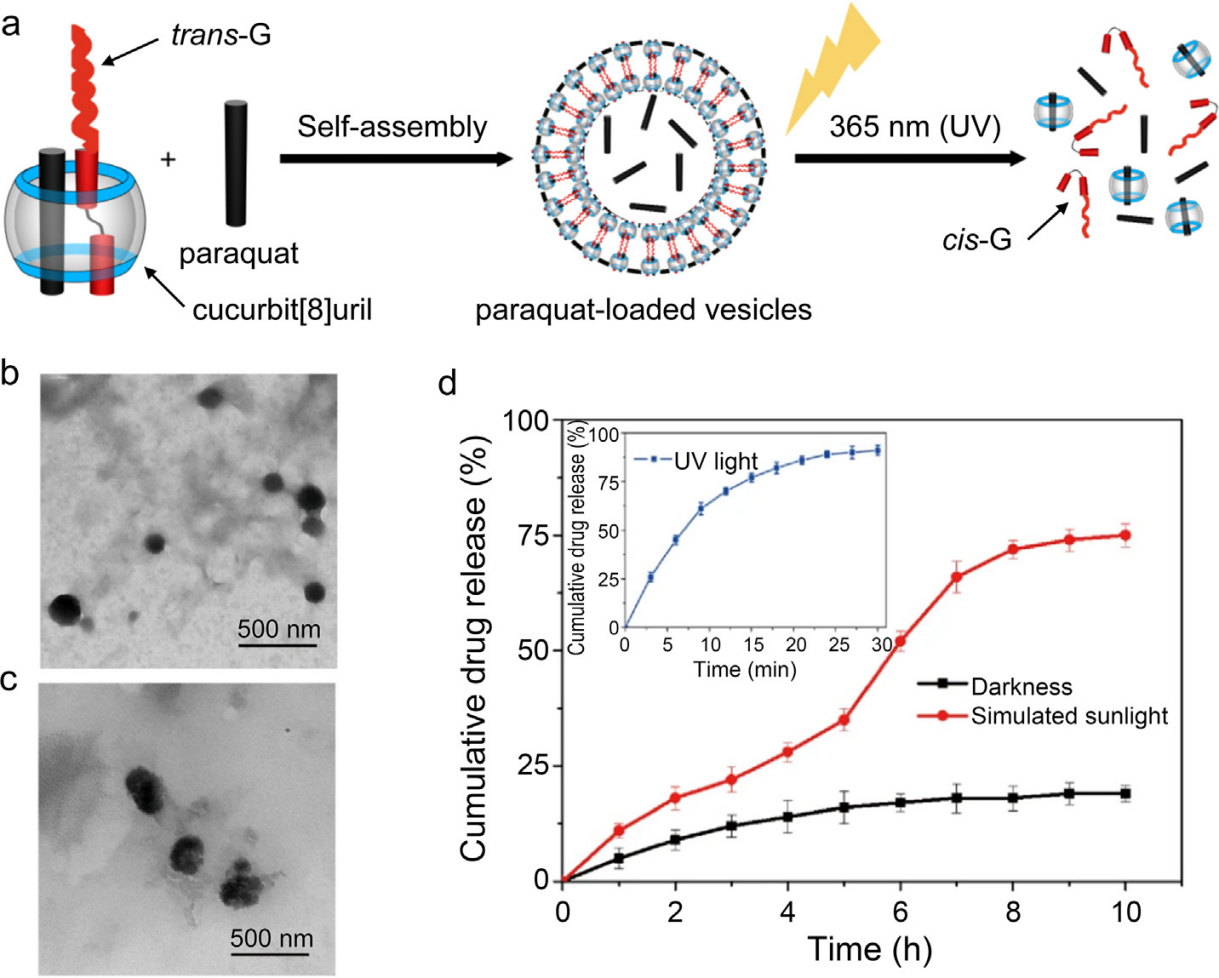
(a) Schematics of the preparation of the paraquat-loaded vesicles and the photoresponsive controlled release of the pesticide AI paraquat upon UV irradiation. (b) The supramolecular vesicles were spherically shaped, and (c) they collapsed and aggregated after UV irradiation. (d) The nanopesticide exhibited a faster release of paraquat under UV light or simulated sunlight than in the dark. Panels a–d were adapted with permission from ref. [144]. Copyright (2018) Springer Nature.

Organic nanocarriers responsive to both infrared and UV light have been developed for the controlled release of pesticide AIs. For example, a controlled-release nanopesticide with NIR and temperature responses was prepared by encapsulating imidacloprid into core–shell PDA@PNIPAm nanocomposites [10]. The response mechanism was as follows: under NIR irradiation, PDA, a photothermal material, can raise the temperature above the LCST of PNIPAm (i.e., 32–33 °C), leading to the collapse of the PNIPAm polymer chains and the release of the imidacloprid molecules encapsulated in the cross-linking networks of PNIPAm. As another notable example, a photoresponsive controlled-release nanoherbicide was facilely prepared by the self-assembly of amphiphilic ternary host-guest complexes between cucurbit[8]uril, paraquat, and the *trans*-isomer of an azobenzene derivative (*trans*-G) in the presence of excess paraquat [144]. Under UV irradiation, the supramolecular vesicles were disintegrated via the *trans*-to-*cis* isomerization of G, releasing the herbicide AI paraquat (Fig. 5). Moreover, the nanopesticide exhibited excellent biosafety, as demonstrated by both *in vitro* cytotoxicity tests with mammalian cell lines and *in vivo* tests with zebrafish and mouse models. Such effective and safe controlled-release nanopesticides hold great promise for applications in green agriculture.

Organic nanocarriers constructed with natural polymers can be degraded by specific enzymes, thus enabling enzyme-responsive, controlled release of pesticide AIs. For preparing controlled-release nanofungicides, the natural polymers employed are typical substrates of lignin-degrading enzymes [154], cellulase [116], and pectinase [79,150], and these enzymes excreted by common pathogenic fungi serve as proper triggers for the release of fungicide AIs. In contrast, nanocarriers for controlled-release insecticides are typically designed to respond to laccase [60,64,73,155], α-amylase [97,109], and proteases [159,160], which are common in the digestive organs of phytophagous insects. Notably, many polymeric nanocarriers contain acidic or basic functional groups and thereby exhibit dual responses to pH and enzymes. For instance, a dual-responsive nanopesticide was prepared by layer-by-layer assembly of chitosan and sodium lignosulfonate onto an alkali lignin-based Pickering emulsion, which achieved the controlled release of avermectin in response to both pH and laccase [64]. Alternatively, nanocarriers composed of enzyme substrates and MOFs that are labile to acid- or base-mediated decomposition also exhibit pH and enzyme dual responsiveness [79,97,116].

### 4.2 Controlled-release pesticides with inorganic nanocarriers

There are only a few examples of stimuli-responsive controlled-release nanopesticides with inorganic nanocarriers, and all were reported prior to 2020 (Table S4). A NIR and pH dual-responsive nanopesticide was prepared by loading chlorpyrifos, an insecticide, onto a nanocomposite of reduced GO (rGO) and Cu_2-*x*_Se [140]. Both the Cu_2-*x*_Se nanocrystals and rGO have a photothermal effect in the NIR range, which can weaken the interaction between chlorpyrifos and the nanocarrier, leading to faster release under NIR irradiation. Moreover, the chlorpyrifos release rate from the rGO-Cu_2-*x*_Se nanocomposite was higher under acidic and basic conditions (e.g., pH 4 and 10) than at neutral pH, due to changes in the strength of the hydrogen bonds between chlorpyrifos and rGO [121]. As another example, a nanopesticide prepared by conjugating kasugamycin onto surface-functionalized ZnO quantum dots via a benzoic–imine covalent bond showed a significantly increased release rate of kasugamycin as pH decreased from 7.4 to 4.5 because the benzoic–imine bond was easily cleaved under acidic conditions [121]. Moreover, the ZnO quantum dots can effectively inhibit the photolysis of kasugamycin under UV irradiation, prolonging its effective lifetime.

### 4.3 Controlled-release pesticides with organic–inorganic composite nanocarriers

A variety of organic–inorganic composite nanocarriers have been investigated for preparing controlled-release pesticides (Table S5). Although organic materials alone are expected to enable the efficient loading of pesticide molecules, it is possible to further increase the loading capacity by incorporating an appropriate amount of inorganic materials with high specific surface areas or hollow structures [83,131,152]. Mesoporous silica nanoparticles with or without a hollow structure are the most commonly used inorganic components for fabricating organic–inorganic composite nanocarriers, owing to their good physical and chemical stability, biocompatibility, and amenability to surface functionalization [via the abundant surface silanol (≡Si–OH) groups]. A range of stimuli-responsive controlled-release characteristics have been achieved using organic–inorganic composite nanocarriers, primarily due to the function of stimuli-responsive polymers [45,57,61,83,103,112,131], though, in some cases, other mechanisms are also involved.

Nanopesticides that are responsive to pH changes have been obtained using various organic–inorganic composite nanocarriers, such as chitosan-diatomite/Fe_3_O_4_ [61], alginate-silica nanoparticles [45], carboxymethyl chitosan-MSNs [55,57], poly(glycidylmethacrylate-*co*-acrylic acid)-hollow mesoporous silica (HMS) [83], polydopamine-attapulgite-calcium alginate [87], and PAA-mesoporous nanoselenium [75]. Moreover, pH-sensitive chelation of metal ions can also be used to design nanopesticides with pH responsiveness. For example, copper ion (Cu^2+^) chelation treatment endowed pH-dependent release patterns of azoxystrobin, a fungicide, from PDA-mesoporous silica nanocomposites. The release rate was higher under weakly acidic (pH 5.8) and alkaline (pH 8.6) conditions than at a neutral pH of 7.2, which was due to the breaking of the Cu^2+^–PDA and Cu^2+^–azoxystrobin coordination bonds under weakly acidic and alkaline conditions, respectively [131].

Temperature response can be achieved by using composite nanocarriers containing temperature-sensitive polymers, and examples include poly(*N*-isopropyl acrylamide-methacrylic acid)-MSN [129], PNIPAm-graphene oxide [132], and poly(*N*-isopropyl acrylamide-*co*-methacrylic acid)-HMS [131]. Alternatively, a novel strategy using a pore-forming agent was employed to achieve the temperature-responsive controlled release of glyphosate, a common herbicide [138]. Glyphosate was loaded in attapulgite@ASO@poly(vinyl alcohol) core–shell nanocomposites together with ammonium bicarbonate, and the as-obtained nanoherbicide exhibited temperature-responsive release behavior. At higher temperatures, ammonium bicarbonate decomposes into carbon dioxide and ammonia gases, producing numerous micro-nanopores in the ASO-poly(vinyl alcohol) shell. Both pore formation and the dissolution of poly(vinyl alcohol) contributed to the accelerated release of glyphosate at a higher temperature.

Enzyme and redox responses can be achieved by nanocarriers containing enzyme substrates or redox-active moieties, respectively (see *Section* 2.4). For example, an enzyme-responsive controlled-release nanopesticide was prepared by loading avermectin in α-cyclodextrin-anchored HMS [152]. The α-cyclodextrin component can be hydrolyzed in the presence of α-amylase, opening channels for the release of the encapsulated insecticide. This enzyme-responsive controlled-release nanopesticide proved more effective in controlling a target pest, the diamondback moth (*Plutella xylostella*), than a commercial formulation of avermectin. As another example, a redox/enzyme dual-responsive controlled-release avermectin nanopesticide was prepared using disulfide bond-linked and starch-coated MSNs as the nanocarriers [148], which could realize controlled release of avermectin in response to glutathione and α-amylase secreted by the insect (*P. xylostella*) through the cleavage of disulfide bonds under reducing conditions and α-amylase-catalyzed degradation of starch.

**Fig. 6:**
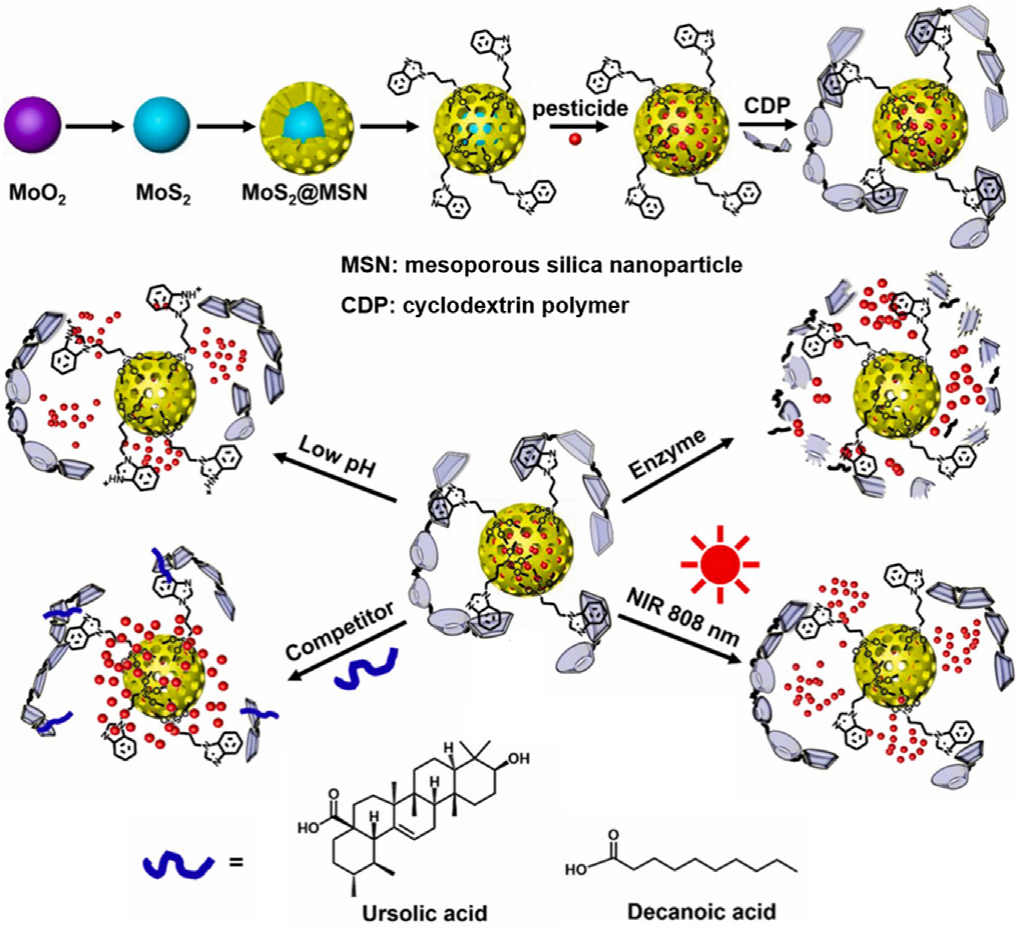
Multi-stimuli-responsive nanopesticide with cyclodextrin polymer-valved benzimidazole-functionalized MoS_2_-embedded mesoporous silica nanoparticles as the nanocarrier, which was responsive to pH, enzyme, near-infrared (NIR), and components of epicuticular waxes (e.g., ursolic acid and decanoic acid) to achieve controlled release of encapsulated pesticide AI. Adapted with permission from ref. [76]. Copyright (2021) Elsevier.

Recently, a multi-stimuli-responsive nanopesticide was developed using cyclodextrin polymer-valved benzimidazole-functionalized MoS_2_-embedded MSN as the nanocarrier, which was responsive to pH, enzyme, and NIR to achieve controlled release of tebuconazole, a fungicide (Fig. 6) [76]. The multivalent supramolecular nanovalves between the cyclodextrin polymer and the benzimidazole moieties were activated at a low pH or in the presence of α-amylase, while MoS_2_ induced the release of tebuconazole under sunlight irradiation via the photothermal effect. Moreover, components of the epicuticular waxes (e.g., ursolic acid and decanoic acid) on the leaves of certain plants triggered the detachment of the cyclodextrin polymer, enabling the release of tebuconazole. It is noted that in designing multi-stimuli-responsive controlled-release nanopesticides, the responses to the individual stimuli should work synergistically or at least not counteract each other [111]; otherwise, an improperly designed nanopesticide may not exhibit the expected controlled release upon multiple stimuli.

## 5 Conclusions and perspectives

Lately, a variety of nanofertilizers and nanopesticides with controlled-release properties have been developed by employing nanocarriers with diverse compositions, structures, and stimuli-response characteristics. A majority of the nanocarriers are composed of organic or organic–inorganic composite materials, whereas inorganic nanocarriers are less common. Most studies on nanofertilizers have focused on pH-responsive nanocarriers, with only a few on temperature- and enzyme-responsive nanocarriers. Compared with nanofertilizers, there are more reports on nanopesticides, which are responsive to a range of environmental or biological stimuli, and many exhibit dual- or triple-responsiveness. These nano-enabled controlled-release fertilizers and pesticides show tremendous potential for enhancing the utilization efficiency of nutrients and pesticide AIs. Despite the fast-growing number of studies on nano-enabled controlled-release fertilizers and pesticides, there are several barriers to their practical applications.

First, future studies should demonstrate the stimuli-responsive controlled-release properties of nanoagrochemicals and their performance for plant growth promotion or pest control under realistic conditions. A majority of the reviewed studies have reported on the release properties in aqueous matrices or methanol/ethanol–water mixtures instead of in soils or other more relevant test media. While release tests in simplified media are efficient for the high-throughput selection of candidate nanocarriers, release tests under realistic conditions are indispensable for accurately correlating the release characteristics of the nanoagrochemicals to their performances. At a minimum, the tested stimulus range must be environmentally relevant. For example, release tests for pH-responsive controlled-release nanofertilizers should be performed within a pH range representative of agricultural soil (e.g., from 5 to 8, or even narrower) or of the crop plant tissues where the nanofertilizers are applied. Only half of the 20 papers on stimuli-responsive nanofertilizers summarized herein (Tables S1 and S2) reported the efficacy of the nanofertilizers in pot experiments, but it is encouraging to observe that data from pot experiments are presented in 6 of the 9 studies published since 2019. It is anticipated that future studies will routinely include pot experiment data, and the results of performance under field conditions are also highly desirable. Moreover, to design nanoagrochemicals with high performance under agriculturally realistic conditions, studies should not only aim at optimizing the controlled-release properties but also take a holistic approach that considers other key characteristics of nanoagrochemicals, such as their adhesion to plant surfaces, dispersity in water, and capability to protect pesticide AIs from photolysis [83,88,112,121,176,177]. Such investigations, which will likely involve processing large datasets with multi-dimensional information and high variability, could be facilitated by emerging data science tools such as machine learning [70,178]. It is noted that pesticides are often applied in mixtures [4], and recently there has been increasing interest in developing controlled-release nanopesticides with more than one AI [56,70,110,147]. The simultaneous loading and coordinated release of multiple pesticide molecules with different structures and properties (e.g., molecular size, hydrophobicity, and water solubility) can be challenging, and techniques capable of real-time monitoring of multiple compounds released from nanomaterials will be instrumental in accelerating such research efforts [179].

Second, unproven economic viability is another major barrier [34]. Although controlled-release nanoagrochemicals are expected to drastically increase the utilization efficiency of nutrients and pesticide AIs, thus reducing their application amounts, the unit costs per mass of nanoagrochemicals are much higher than those of their traditional counterparts, offsetting their potential economic advantage. Stimuli-responsive controlled-release nanocarriers have been extensively studied for biomedical applications (e.g., precision drug delivery) [[180], [181], [182], [183]], where efficacy is a far more important factor for consideration than cost. However, with a much smaller profit margin in agriculture, the cost is a major concern in developing nanoagrochemicals. The cost is expected to decrease after scale-up production, but cost-effectiveness should be considered in the initial research and design stages by selecting suitable raw materials and procedures.

Finally, regulatory and public concerns constitute barriers that must be lifted before these nanoagrochemicals can be applied to agricultural lands on a large scale [1,11,[184], [185], [186]]. Considering that nanofertilizers and nanopesticides may be applied or translocated to the edible parts of crop plants [187], it is desirable to use non-toxic and biodegradable materials for constructing the nanocarriers. Moreover, it is necessary to ensure that nanofertilizers and nanopesticides, as potential emerging contaminants, have negligible adverse effects on non-target organisms at doses required for agricultural production [[188], [189], [190]]. So far, only 21 of the studies summarized herein, among which 19 were published between 2021 and 2023, have tested the ecotoxicity of the nanoagrochemicals to non-target organisms, including zebrafish [64,69,73,99,102,104,119,128,134,137,139,144,150], water fleas [107,116], honeybees [77], ladybugs [155], earthworms [82,97], nematodes [143], and other soil microorganisms [107,116]. Notably, novel materials such as MOFs have been actively investigated as stimuli-responsive nanocarriers for controlled-release nanopesticides. However, their environmental health implications as potential emerging contaminants remain largely unknown. Although the MOFs that have been explored for fabricating nanopesticides so far contain non-toxic metals (e.g., Fe, Zn, and Zr), the toxicities of the MOFs as chemical entities as well as the organic linkers released after decomposition of the MOFs require careful evaluation. Similarly, other emerging materials should also be subjected to this scrutiny if they are to be explored for preparing nanoagrochemicals.

In summary, to obtain high-efficiency and safe nanofertilizers and nanopesticides, it is advisable to adopt a comprehensive design strategy that not only optimizes the controlled-release properties and lowers the costs but also considers other critical factors, including their interaction mechanisms with crop plants [[191], [192], [193]], pests, and non-target organisms [188,189], as well as their transformation and fate in the agricultural environment [194].

## Author contributions

M. S. and S. L.: data curation, investigation, visualization, writing–original draft. C. J.: conceptualization, funding acquisition, investigation, supervision, writing–original draft, writing–review & editing. T. Z. and W. C.: funding acquisition, supervision, writing–review & editing.

## Declaration of competing interests

There are no conflicts of interest to declare.

## References

[1] Lowry G.V., Avellan A., Gilbertson L.M. (2019). Opportunities and challenges for nanotechnology in the agri-tech revolution. Nat. Nanotechnol..

[2] Rosenzweig C., Elliott J., Deryng D., Ruane A.C., Muller C., Arneth A., Boote K.J., Folberth C. (2014). Assessing agricultural risks of climate change in the 21st century in a global gridded crop model intercomparison. Proc. Natl. Acad. Sci. USA.

[3] Mali H., Shah C., Raghunandan B.H., Prajapati A.S., Patel D.H., Trivedi U., Subramanian R.B. (2023). Organophosphate pesticides an emerging environmental contaminant: pollution, toxicity, bioremediation progress, and remaining challenges. J. Environ. Sci..

[4] Schaeffer A., Wijntjes C. (2022). Changed degradation behavior of pesticides when present in mixtures. Eco Environ. Health.

[5] Gilbertson L.M., Pourzahedi L., Laughton S., Gao X., Zimmerman J.B., Theis T.L., Westerhoff P., Lowry G.V. (2020). Guiding the design space for nanotechnology to advance sustainable crop production. Nat. Nanotechnol..

[6] Urso J.H., Gilbertson L.M. (2018). Atom conversion efficiency: a new sustainability metric applied to nitrogen and phosphorus use in agriculture. ACS Sustainable Chem. Eng..

[7] Zhao X., Cui H., Wang Y., Sun C., Cui B., Zeng Z. (2018). Development strategies and prospects of nano-based smart pesticide formulation. J. Agric. Food Chem..

[8] Zhang M., Gao B., Chen J.J., Li Y.C., Creamer A.E., Chen H. (2014). Slow-release fertilizer encapsulated by graphene oxide films. Chem. Eng. J..

[9] Kottegoda N., Sandaruwan C., Priyadarshana G., Siriwardhana A., Rathnayake U.A., Arachchige D.M.B., Kumarasinghe A.R., Dahanayake D. (2017). Urea-hydroxyapatite nanohybrids for slow release of nitrogen. ACS Nano.

[10] Xu X.H., Bai B., Wang H.L., Suo Y.R. (2017). A near-infrared and temperature-responsive pesticide release platform through core-shell polydopamine@PNIPAm nanocomposites. ACS Appl. Mater. Interfaces.

[11] Hofmann T., Lowry G.V., Ghoshal S., Tufenkji N., Brambilla D., Dutcher J.R., Gilbertson L.M., Giraldo J.P. (2020). Technology readiness and overcoming barriers to sustainably implement nanotechnology-enabled plant agriculture. Nat. Food.

[12] Fu L., Wang Z., Dhankher O.P., Xing B. (2019). Nanotechnology as a new sustainable approach for controlling crop diseases and increasing agricultural production. J. Exp. Bot..

[13] Giraldo J.P., Wu H.H., Newkirk G.M., Kruss S. (2019). Nanobiotechnology approaches for engineering smart plant sensors. Nat. Nanotechnol..

[14] Ghorbanpour M., Mohammadi H., Kariman K. (2020). Nanosilicon-based recovery of barley (*Hordeum vulgare*) plants subjected to drought stress. Environ. Sci.: Nano.

[15] Rossi L., Zhang W., Ma X. (2017). Cerium oxide nanoparticles alter the salt stress tolerance of *Brassica napus* L. by modifying the formation of root apoplastic barriers. Environ. Pollut..

[16] Wan J., Wang R., Bai H., Wang Y., Xu J. (2020). Comparative physiological and metabolomics analysis reveals that single-walled carbon nanohorns and ZnO nanoparticles affect salt tolerance in *Sophora alopecuroides*. Environ. Sci.: Nano.

[17] Asadishad B., Chahal S., Akbari A., Cianciarelli V., Azodi M., Ghoshal S., Tufenkji N. (2018). Amendment of agricultural soil with metal nanoparticles: effects on soil enzyme activity and microbial community composition. Environ. Sci. Technol..

[18] Kah M., Kookana R.S., Gogos A., Bucheli T.D. (2018). A critical evaluation of nanopesticides and nanofertilizers against their conventional analogues. Nat. Nanotechnol..

[19] Kah M., Tufenkji N., White J.C. (2019). Nano-enabled strategies to enhance crop nutrition and protection. Nat. Nanotechnol..

[20] Adisa I.O., Pullagurala V.L.R., Peralta-Videa J.R., Dimkpa C.O., Elmer W.H., Gardea-Torresdey J.L., White J.C. (2019). Recent advances in nano-enabled fertilizers and pesticides: a critical review of mechanisms of action. Environ. Sci.: Nano.

[21] Chaud M., Souto E.B., Zielinska A., Severino P., Batain F., Oliveira-Junior J., Alves T. (2021). Nanopesticides in agriculture: benefits and challenge in agricultural productivity, toxicological risks to human health and environment. Toxics.

[22] Ashraf H., Anjum T., Riaz S., Ahmad I.S., Irudayaraj J., Javed S., Qaiser U., Naseem S. (2021). Inhibition mechanism of green-synthesized copper oxide nanoparticles from cassia fistula towards *Fusarium oxysporum* by boosting growth and defense response in tomatoes. Environ. Sci.: Nano.

[23] Zhang X., Xu Z., Qian X., Lin D., Zeng T., Filser J., Li L., Kah M. (2020). Assessing the impacts of Cu(OH)_2_ nanopesticide and ionic copper on the soil enzyme activity and bacterial community. J. Agric. Food Chem..

[24] Ma C., Borgatta J., Hudson B.G., Tamijani A.A., De La Torre-Roche R., Zuverza-Mena N., Shen Y., Elmer W. (2020). Advanced material modulation of nutritional and phytohormone status alleviates damage from soybean sudden death syndrome. Nat. Nanotechnol..

[25] Shang H., Ma C., Li C., White J.C., Polubesova T., Chefetz B., Xing B. (2020). Copper sulfide nanoparticles suppress *Gibberella fujikuroi* infection in rice (*Oryza sativa* L.) by multiple mechanisms: contact-mortality, nutritional modulation and phytohormone regulation. Environ. Sci.: Nano.

[26] Pradhan S., Barik S., Goswami A. (2019). Assessment of photo-modulation, nutrient-use efficiency and toxicity of iron nanoparticles in *Vigna radiata*. Environ. Sci.: Nano.

[27] Liu Y., Wu T., White J.C., Lin D. (2021). A new strategy using nanoscale zero-valent iron to simultaneously promote remediation and safe crop production in contaminated soil. Nat. Nanotechnol..

[28] Wang C., Cheng T., Liu H., Zhou F., Zhang J., Zhang M., Liu X., Shi W. (2021). Nano-selenium controlled cadmium accumulation and improved photosynthesis in indica rice cultivated in lead and cadmium combined paddy soils. J. Environ. Sci..

[29] Dimkpa C.O., Singh U., Adisa I.O., Bindraban P.S., Elmer W.H., Gardea-Torresdey J.L., White J.C. (2018). Effects of manganese nanoparticle exposure on nutrient acquisition in wheat (*Triticum aestivum* L.). Agronomy.

[30] Ma C.X., Li Q.Q., Jia W.L., Shang H.P., Zhao J., Hao Y., Li C.Y., Tomko M. (2021). Role of nanoscale hydroxyapatite in disease suppression of fusarium-infected tomato. Environ. Sci. Technol..

[31] Cai L., Liu C., Fan G., Liu C., Sun X. (2019). Preventing viral disease by ZnONPs through directly deactivating TMV and activating plant immunity in *Nicotiana benthamiana*. Environ. Sci.: Nano.

[32] Elmer W.H., White J.C. (2016). The use of metallic oxide nanoparticles to enhance growth of tomatoes and eggplants in disease infested soil or soilless medium. Environ. Sci.: Nano.

[33] Camara M.C., Campos E.V.R., Monteiro R.A., Pereira A.D.S., Proenca P.L.D., Fraceto L.F. (2019). Development of stimuli-responsive nano-based pesticides: emerging opportunities for agriculture. J. Nanobiotechnol..

[34] Dimkpa C.O., Bindraban P.S. (2018). Correction to nanofertilizers: new products for the industry?. J. Agric. Food Chem..

[35] Vejan P., Khadiran T., Abdullah R., Ahmad N. (2021). Controlled release fertilizer: a review on developments, applications and potential in agriculture. J. Contr. Release.

[36] Li N., Sun C., Jiang J., Wang A., Wang C., Shen Y., Huang B., An C., Wang (2021). Advances in controlled-release pesticide formulations with improved efficacy and targetability. J. Agric. Food Chem..

[37] Xiao D., Wu H., Zhang Y., Kang J., Dong A., Liang W. (2022). Advances in stimuli-responsive systems for pesticides delivery: recent efforts and future outlook. J. Contr. Release.

[38] Tao R., You C., Qu Q., Zhang X., Deng Y., Ma W., Huang C. (2023). Recent advances in the design of controlled- and sustained-release micro/nanocarriers of pesticide. Environ. Sci.: Nano.

[39] Rindt D.W., Blouin G.M., Getsinger J.G. (1968). Sulfur coating on nitrogen fertilizer to reduce dissolution rate. J. Agric. Food Chem..

[40] Allen S.E., Mays D.A. (1971). Sulfur-coated fertilizers for controlled release: agronomic evaluation. J. Agric. Food Chem..

[41] Liang R., Liu M. (2006). Preparation and properties of a double-coated slow-release and water-retention urea fertilizer. J. Agric. Food Chem..

[42] Ni B., Liu M., Lu S., Xie L., Wang Y. (2011). Environmentally friendly slow-release nitrogen fertilizer. J. Agric. Food Chem..

[43] Yang Y., Tong Z., Geng Y., Li Y., Zhang M. (2013). Biobased polymer composites derived from corn stover and feather meals as double-coating materials for controlled-release and water-retention urea fertilizers. J. Agric. Food Chem..

[44] Xie J., Yang Y., Gao B., Wan Y., Li Y.C., Cheng D., Xiao T., Li K. (2019). Magnetic-sensitive nanoparticle self-assembled superhydrophobic biopolymer-coated slow-release fertilizer: fabrication, enhanced performance, and mechanism. ACS Nano.

[45] Chen K., Yu G., He F., Zhou Q., Xiao D., Li J., Feng Y. (2017). A pH-responsive emulsion stabilized by alginate-grafted anisotropic silica and its application in the controlled release of λ-cyhalothrin. Carbohydr. Polym..

[46] Zheng D., Wang K., Bai B., Hu N., Wang H. (2022). Swelling and glyphosate-controlled release behavior of multi-responsive alginate-*g*-P(NIPAm-*co*-NDEAm)-based hydrogel. Carbohydr. Polym..

[47] Kumar S., Chauhan N., Gopal M., Kumar R., Dilbaghi N. (2015). Development and evaluation of alginate-chitosan nanocapsules for controlled release of acetamiprid. Int. J. Biol. Macromol..

[48] Zha X., Hou X., Li Q., Nan H., Ge F., Liu Y., Li F., Zhang D., Tian J. (2022). Loading glyphosate in attapulgite and sodium alginate hydrogels to construct pH-responsive controlled release microsphere for enhanced soil sustained release. ACS Agric. Sci. Technol..

[49] Rashidzadeh A., Olad A., Salari D., Reyhanitabar A. (2014). On the preparation and swelling properties of hydrogel nanocomposite based on sodium alginate-*g*-Poly (acrylic acid-*co*-acrylamide)/Clinoptilolite and its application as slow release fertilizer. J. Polym. Res..

[50] Rashidzadeh A., Olad A. (2014). Slow-released NPK fertilizer encapsulated by NaAlg-*g*-poly(AA-*co*-AAm)/MMT superabsorbent nanocomposite. Carbohydr. Polym..

[51] Shaghaleh H., Alhaj Hamoud Y., Xu X., Wang S., Liu H. (2022). A pH-responsive/sustained release nitrogen fertilizer hydrogel based on aminated cellulose nanofiber/cationic copolymer for application in irrigated neutral soils. J. Clean. Prod..

[52] Lohmousavi S.M., Abad H.H.S., Noormohammadi G., Delkhosh B. (2020). Synthesis and characterization of a novel controlled release nitrogen-phosphorus fertilizer hybrid nanocomposite based on banana peel cellulose and layered double hydroxides nanosheets. Arab. J. Chem..

[53] Zhao M., Zhou H., Chen L., Hao L., Chen H., Zhou X. (2020). Carboxymethyl chitosan grafted trisiloxane surfactant nanoparticles with pH sensitivity for sustained release of pesticide. Carbohydr. Polym..

[54] Hou X., Pan Y., Xiao H., Liu J. (2019). Controlled release of agrochemicals using pH and redox dual-responsive cellulose nanogels. J. Agric. Food Chem..

[55] Song S., Wang Y., Xie J., Sun B., Zhou N., Shen H., Shen J. (2019). Carboxymethyl chitosan modified carbon nanoparticle for controlled emamectin benzoate delivery: improved solubility, pH-responsive release, and sustainable pest control. ACS Appl. Mater. Interfaces.

[56] Dong J., Liu X., Chen Y., Yang W., Du X. (2021). User-safe and efficient chitosan-gated porous carbon nanopesticides and nanoherbicides. J. Colloid Interface Sci..

[57] Xu C., Cao L., Zhao P., Zhou Z., Cao C., Li F., Huang Q. (2018). Emulsion-based synchronous pesticide encapsulation and surface modification of mesoporous silica nanoparticles with carboxymethyl chitosan for controlled azoxystrobin release. Chem. Eng. J..

[58] Li G.-B., Wang J., Kong X.-P. (2020). Coprecipitation-based synchronous pesticide encapsulation with chitosan for controlled spinosad release. Carbohydr. Polym..

[59] Chauhan N., Dilbaghi N., Gopal M., Kumar R., Kim K.H., Kumar S. (2017). Development of chitosan nanocapsules for the controlled release of hexaconazole. Int. J. Biol. Macromol..

[60] Yu X., Wang J., Li X., Ma S., Zhu W., Wang H. (2023). Dual-responsive microcapsules with tailorable shells from oppositely charged biopolymers for precise pesticide release. Mater. Adv..

[61] Xiang Y.B., Zhang G.L., Chi Y., Cai D.Q., Wu Z.Y. (2017). Fabrication of a controllable nanopesticide system with magnetic collectability. Chem. Eng. J..

[62] Zhou Y., Wu J., Zhou J., Lin S., Cheng D. (2022). pH-responsive release and washout resistance of chitosan-based nano-pesticides for sustainable control of plumeria rust. Int. J. Biol. Macromol..

[63] Zhao M., Li P., Zhou H., Hao L., Chen H., Zhou X. (2022). pH/redox dual responsive from natural polymer-based nanoparticles for on-demand delivery of pesticides. Chem. Eng. J..

[64] Yu X., Li X., Ma S., Wang Y., Zhu W., Wang H. (2023). Biomass-based, interface tunable, and dual-responsive pickering emulsions for smart release of pesticides. Adv. Funct. Mater..

[65] Wen P., Wu Z.S., He Y.H., Ye B.C., Han Y.J., Wang J., Guan X.Y. (2016). Microwave-assisted synthesis of a semi-interpenetrating polymer network slow-release nitrogen fertilizer with water absorbency from cotton stalks. ACS Sustainable Chem. Eng..

[66] Chen H., Zhi H., Liang J., Yu M., Cui B., Zhao X., Sun C., Wang Y. (2021). Development of leaf-adhesive pesticide nanocapsules with pH-responsive release to enhance retention time on crop leaves and improve utilization efficiency. J. Mater. Chem. B.

[67] Song S., Wan M., Luo Y., Shen H., Shen J. (2022). Carboxymethyl chitosan-modified graphene oxide as a multifunctional vector for deltamethrin delivery and pH-responsive controlled release, enhanced leaf affinity, and improved mosquito-killing activity. Langmuir.

[68] Tong Y., Shao L., Li X., Lu J., Sun H., Xiang S., Zhang Z., Wu Y. (2018). Adhesive and stimulus-responsive polydopamine-coated graphene oxide system for pesticide-loss control. J. Agric. Food Chem..

[69] Yang L., Chen H., Yan W., Huang S., Cheng D., Xu H., Zhang Z. (2022). A pH- and redox-stimulated responsive hollow mesoporous silica for triggered delivery of fungicides to control downy mildew of *Luffa cylindrica*. Pest Manag. Sci..

[70] Ji Y., Ma S., Lv S., Wang Y., Lu S., Liu M. (2021). Nanomaterials for targeted delivery of agrochemicals by an all-in-one combination strategy and deep learning. ACS Appl. Mater. Interfaces.

[71] Chen L., Zhou X., Lin G., Chen H., Hao L., Zhou H. (2019). Synthesis of pH-responsive isolated soy protein/carboxymethyl chitosan microspheres for sustained pesticide release. J. Appl. Polym. Sci..

[72] Li T., Lu S.Y., Yan J., Bai X., Gao C.M., Liu M.Z. (2019). An environment-friendly fertilizer prepared by layer-by-layer self-assembly for pH-responsive nutrient release. ACS Appl. Mater. Interfaces.

[73] Zhang D.X., Du J., Wang R., Luo J., Jing T.F., Li B.X., Mu W., Liu F. (2021). Core/shell dual-responsive nanocarriers via iron-mineralized electrostatic self-assembly for precise pesticide delivery. Adv. Funct. Mater..

[74] Olad A., Gharekhani H., Mirmohseni A., Bybordi A. (2018). Superabsorbent nanocomposite based on maize bran with integration of water-retaining and slow-release NPK fertilizer. Adv. Polym. Technol..

[75] Liu J., Zhu X., Chen X., Liu Y., Gong Y., Yuan G., Liu J., Chen L. (2020). Defense and inhibition integrated mesoporous nanoselenium delivery system against tomato gray mold. Environ. Sci.: Nano.

[76] Dong J., Chen W., Qin D., Chen Y., Li J., Wang C., Yu Y., Feng J. (2021). Cyclodextrin polymer-valved MoS_2_-embedded mesoporous silica nanopesticides toward hierarchical targets via multidimensional stimuli of biological and natural environments. J. Hazard Mater..

[77] Hou R., Li C., Tan Y., Wang Y., Huang S., Zhao C., Zhang Z. (2023). Eco-friendly *O*-carboxymethyl chitosan base chlorfenapyr nanopesticide for effective pest control and reduced toxicity to honey bees. Int. J. Biol. Macromol..

[78] Feng C., Lü S., Gao C., Wang X., Xu X., Bai X., Gao N., Liu M. (2015). “Smart” fertilizer with temperature- and pH-responsive behavior via surface-initiated polymerization for controlled release of nutrients. ACS Sustainable Chem. Eng..

[79] Tang J., Ding G., Niu J., Zhang W., Tang G., Liang Y., Fan C., Dong H. (2019). Preparation and characterization of tebuconazole metal-organic framework-based microcapsules with dual-microbicidal activity. Chem. Eng. J..

[80] Zhang Y., Yan J., Avellan A., Gao X., Matyjaszewski K., Tilton R.D., Lowry G.V. (2020). Temperature- and pH-responsive star polymers as nanocarriers with potential for in vivo agrochemical delivery. ACS Nano.

[81] Zhang Y., Fu L., Martinez M.R., Sun H., Nava V., Yan J., Ristroph K., Averick S.E. (2023). Temperature-responsive bottlebrush polymers deliver a stress-regulating agent in vivo for prolonged plant heat stress mitigation. ACS Sustainable Chem. Eng..

[82] Wu T., Zhao K., Zhang C., Zhong T., Li Z., Bao Z., Gao Y., Du F. (2022). Promising delivery platform for smart pest control with high water-retaining capacity. ACS Appl. Mater. Interfaces.

[83] Gao Y., Zhang Y., He S., Xiao Y., Qin X., Zhang Y., Li D., Ma H. (2019). Fabrication of a hollow mesoporous silica hybrid to improve the targeting of a pesticide. Chem. Eng. J..

[84] Wu H., Gong L., Zhang X., He F., Li Z. (2021). Bifunctional porous polyethyleneimine-grafted lignin microspheres for efficient adsorption of 2,4-dichlorophenoxyacetic acid over a wide pH range and controlled release. Chem. Eng. J..

[85] Bortolin A., Aouada F.A., Mattoso L.H.C., Ribeiro C. (2013). Nanocomposite PAAm/methyl cellulose/montmorillonite hydrogel: evidence of synergistic effects for the slow release of fertilizers. J. Agric. Food Chem..

[86] Zhang G., Zhou L., Cai D., Wu Z. (2018). Anion-responsive carbon nanosystem for controlling selenium fertilizer release and improving selenium utilization efficiency in vegetables. Carbon.

[87] Xiang Y., Zhang G., Chen C., Liu B., Cai D., Wu Z. (2018). Fabrication of a pH-responsively controlled-release pesticide using an attapulgite-based hydrogel. ACS Sustainable Chem. Eng..

[88] Sharma S., Singh B., Bindra P., Panneerselvam P., Dwivedi N., Senapati A., Adholeya A., Shanmugam V. (2021). Triple-smart eco-friendly chili anthracnose control agro-nanocarrier. ACS Appl. Mater. Interfaces.

[89] Olad A., Zebhi H., Salari D., Mirmohseni A., Tabar A.R. (2018). Slow-release NPK fertilizer encapsulated by carboxymethyl cellulose-based nanocomposite with the function of water retention in soil. Mater. Sci. Eng. C.

[90] Olad A., Zebhi H., Salari D., Mirmohseni A., Tabar A.R. (2018). Water retention and slow release studies of a salep-based hydrogel nanocomposite reinforced with montmorillonite clay. New J. Chem..

[91] Yearla S.R., Padmasree K. (2016). Exploitation of subabul stem lignin as a matrix in controlled release agrochemical nanoformulations: a case study with herbicide diuron. Environ. Sci. Pollut. Res..

[92] Song S., Wan M., Feng W., Tian Y., Jiang X., Luo Y., Shen J. (2022). Environmentally friendly Zr-based MOF for pesticide delivery: ultrahigh loading capacity, pH-responsive release, improved leaf affinity, and enhanced antipest activity. Langmuir.

[93] Castro G.F.d., Mattiello E.M., Ferreira J.A., Zotarelli L., Tronto J. (2020). Synthesis, characterization and agronomic use of alginate microspheres containing layered double hydroxides intercalated with borate. New J. Chem..

[94] Su C., Ji Y., Liu S., Gao S., Cao S., Xu X., Zhou C., Liu Y. (2020). Fluorescence-labeled abamectin nanopesticide for comprehensive control of pinewood nematode and monochamus alternatus hope. ACS Sustainable Chem. Eng..

[95] Zhao M., Zhou H., Chen L., Hao L., Chen H., Zhou X. (2020). Carboxymethyl chitosan grafted trisiloxane surfactant nanoparticles with pH sensitivity for sustained release of pesticide. Carbohydr. Polym..

[96] Kaziem A.E., Gao Y., He S., Li J. (2017). Synthesis and insecticidal activity of enzyme-triggered functionalized hollow mesoporous silica for controlled release. J. Agric. Food Chem..

[97] Zhang X., He Y., Yuan Z., Shen G., Zhang Z., Niu J., He L., Wang J. (2023). A pH- and enzymatic-responsive nanopesticide to control pea aphids and reduce toxicity for earthworms. Sci. Total Environ..

[98] Liang W., Xie Z., Cheng J., Xiao D., Xiong Q., Wang Q., Zhao J., Gui W. (2021). A light-triggered pH-responsive metal–organic framework for smart delivery of fungicide to control sclerotinia diseases of oilseed rape. ACS Nano.

[99] Wu L.T., Pan H., Huang W.L., Wang M.J., Hu Z.X., Zhang F. (2022). Self-assembled degradable iron-doped mesoporous silica nanoparticles for the smart delivery of prochloraz to improve plant protection and reduce environmental impact. Environ. Technol. Innovat..

[100] Wang Y., Peng Z., Yang Y., Li Z., Wen Y., Liu M., Li S., Su L. (2022). Auricularia auricula biochar supported γ-FeOOH nanoarrays for electrostatic self-assembly and pH-responsive controlled release of herbicide and fertilizer. Chem. Eng. J..

[101] Wu H., Hu P., Xu Y., Xiao C., Chen Z., Liu X., Jia J., Xu H. (2021). Phloem delivery of fludioxonil by plant amino acid transporter-mediated polysuccinimide nanocarriers for controlling fusarium wilt in banana. J. Agric. Food Chem..

[102] Teng G., Chen C., Jing N., Chen C., Duan Y., Zhang L., Wu Z., Zhang J. (2023). Halloysite nanotubes-based composite material with acid/alkali dual pH response and foliar adhesion for smart delivery of hydrophobic pesticide. Chem. Eng. J..

[103] Hao L., Gong L., Chen L., Guan M., Zhou H., Qiu S., Wen H., Chen H. (2020). Composite pesticide nanocarriers involving functionalized boron nitride nanoplatelets for pH-responsive release and enhanced UV stability. Chem. Eng. J..

[104] Shan P., Lu Y., Lu W., Yin X., Liu H., Li D., Lian X., Wang W. (2022). Biodegradable and light-responsive polymeric nanoparticles for environmentally safe herbicide delivery. ACS Appl. Mater. Interfaces.

[105] Berber M.R., Hafez I.H. (2018). Synthesis of a new nitrate-fertilizer form with a controlled release behavior via an incorporation technique into a clay material. Bull. Environ. Contam. Toxicol..

[106] Xu C., Shan Y., Bilal M., Xu B., Cao L., Huang Q. (2020). Copper ions chelated mesoporous silica nanoparticles via dopamine chemistry for controlled pesticide release regulated by coordination bonding. Chem. Eng. J..

[107] Kaziem A.E., Yang L., Lin Y., Kazem A.E., Xu H., Zhang Z. (2021). Pathogenic invasion-responsive carrier based on mesoporous silica/β-glucan nanoparticles for smart delivery of fungicides. ACS Sustainable Chem. Eng..

[108] Gao Y., Liang Y., Zhou Z., Yang J., Tian Y., Niu J., Tang G., Tang J. (2021). Metal-organic framework nanohybrid carrier for precise pesticide delivery and pest management. Chem. Eng. J..

[109] Liang Y., Wang S., Jia H., Yao Y., Song J., Yang W., Cao Y., Zhu F. (2022). pH/redox/α-amylase triple responsive metal-organic framework composites for pest management and plant growth promotion. Microporous Mesoporous Mater..

[110] Wang Y., Ma S., Yang X., Li Y., Lü S. (2022). Facile synthesis of the dual pesticide-loaded metal–organic framework hybrid for pH-responsive release. ACS Agric. Sci. Technol..

[111] Dong J., Chen W., Feng J., Liu X., Xu Y., Wang C., Yang W., Du X. (2021). Facile, smart, and degradable metal–organic framework nanopesticides gated with FeIII-tannic acid networks in response to seven biological and environmental stimuli. ACS Appl. Mater. Interfaces.

[112] Feng P., Chen J., Fan C., Huang G., Yu Y., Wu J., Lin B. (2020). An eco-friendly MIL-101@CMCS double -coated dinotefuran for long-acting active release and sustainable pest control. J. Clean. Prod..

[113] Shan Y., Xu C., Zhang H., Chen H., Bilal M., Niu S., Cao L., Huang Q. (2020). Polydopamine-modified metal–organic frameworks, NH_2_-Fe-MIL-101, as pH-sensitive nanocarriers for controlled pesticide release. Nanomaterials.

[114] Liu G., Lin G., Tan M., Zhou H., Chen H., Xu H., Zhou X. (2019). Hydrazone-linked soybean protein isolate-carboxymethyl cellulose conjugates for pH-responsive controlled release of pesticides. Polym. J..

[115] Huang Y., Yang Y., Liang B., Lu S., Yuan X., Jia Z., Liu J., Liu Y. (2023). Green nanopesticide: pH-responsive eco-friendly pillar[5]arene-modified selenium nanoparticles for smart delivery of carbendazim to suppress sclerotinia diseases. ACS Appl. Mater. Interfaces.

[116] Ma Y., Yu M., Wang Y., Pan S., Sun X., Zhao R., Sun Z., Gao R. (2023). A pH/cellulase dual stimuli-responsive cellulose-coated metal–organic framework for eco-friendly fungicide delivery. Chem. Eng. J..

[117] Ma S., Ji Y., Dong Y., Chen S., Wang Y., Lu S. (2021). An environmental-friendly pesticide-fertilizer combination fabricated by in-situ synthesis of ZIF-8. Sci. Total Environ..

[118] Aguliar Perez K.M., Alagoz Y., Maatouk B., Wang J.Y., Berqdar L., Qutub S., Jamil M., AlNasser S. (2023). Biomimetic mineralization for smart biostimulant delivery and crop micronutrients fortification. Nano Lett..

[119] Ren L., Zhao J., Li W., Li Q., Zhang D., Fang W., Yan D., Li Y. (2022). Site-specific controlled-release imidazolate framework-8 for dazomet smart delivery to improve the effective utilization rate and reduce biotoxicity. J. Agric. Food Chem..

[120] Liang W., Cheng J., Zhang J., Xiong Q., Jin M., Zhao J. (2022). pH-responsive on-demand alkaloids release from core-shell ZnO@ZIF-8 nanosphere for synergistic control of bacterial wilt disease. ACS Nano.

[121] Liang Y., Duan Y., Fan C., Dong H., Yang J., Tang J., Tang G., Wang W. (2019). Preparation of kasugamycin conjugation based on ZnO quantum dots for improving its effective utilization. Chem. Eng. J..

[122] Wang M., Zhang G.L., Zhou L.L., Wang D.F., Zhong N.Q., Cai D.Q., Wu Z.Y. (2016). Fabrication of pH-controlled-release ferrous foliar fertilizer with high adhesion capacity based on nanobiomaterial. ACS Sustainable Chem. Eng..

[123] Wen H., Zhou H., Hao L., Chen H., Xu H., Zhou X. (2020). Enzyme cum pH dual-responsive controlled release of avermectin from functional polydopamine microcapsules. Colloids Surf., B.

[124] Shan Y., Cao L., Xu C., Zhao P., Cao C., Li F., Xu B., Huang Q. (2019). Sulfonate-functionalized mesoporous silica nanoparticles as carriers for controlled herbicide diquat dibromide release through electrostatic interaction. Int. J. Mol. Sci..

[125] Cao L., Zhou Z., Niu S., Cao C., Li X., Shan Y., Huang Q. (2018). Positive-charge functionalized mesoporous silica nanoparticles as nanocarriers for controlled 2,4-dichlorophenoxy acetic acid sodium salt release. J. Agric. Food Chem..

[126] Chi Y., Zhang G.L., Xiang Y.B., Cai D.Q., Wu Z.Y. (2018). Fabrication of reusable temperature-controlled-released fertilizer using a palygorskite-based magnetic nanocomposite. Appl. Clay Sci..

[127] Shang H., Yang X., Liu H. (2023). Temperature-responsive hydrogel prepared from carboxymethyl cellulose-stabilized N-vinylcaprolactam with potential for fertilizer delivery. Carbohydr. Polym..

[128] Shen Y., An C., Jiang J., Huang B., Li N., Sun C., Wang C., Zhan S. (2022). Temperature-dependent nanogel for pesticide smart delivery with improved foliar dispersion and bioactivity for efficient control of multiple pests. ACS Nano.

[129] Yang J., Feng J., He K., Chen Z., Chen W., Cao H., Yuan S. (2021). Preparation of thermosensitive buprofezin-loaded mesoporous silica nanoparticles by the sol–gel method and their application in pest control. Pest Manag. Sci..

[130] Qiao D., Li J., Zhang S., Yang X. (2022). Controlled release fertilizer with temperature-responsive behavior coated using polyether polyol (PPG)/polycaprolactone (PCL) blend-based polyurethane performs smart nutrient release. Mater. Today Chem..

[131] Gao Y., Xiao Y., Mao K., Qin X., Zhang Y., Li D., Zhang Y., Li J. (2020). Thermoresponsive polymer-encapsulated hollow mesoporous silica nanoparticles and their application in insecticide delivery. Chem. Eng. J..

[132] Wang Y., Song S., Chu X., Feng W., Li J., Huang X., Zhou N., Shen J. (2021). A new temperature-responsive controlled-release pesticide formulation – poly(*N*-isopropylacrylamide) modified graphene oxide as the nanocarrier for lambda-cyhalothrin delivery and their application in pesticide transportation. Colloids Surf., A.

[133] Xu J., Li H., Niu Y. (2023). Synthesis of a temperature sensitive graft carbon dioxide-based copolymer and its evaluation as a nano drug carrier. Polym. Adv. Technol..

[134] Du Q., Chen L., Ding X., Cui B., Chen H., Gao F., Wang Y., Cui H. (2022). Development of emamectin benzoate-loaded liposome nano-vesicles with thermo-responsive behavior for intelligent pest control. J. Mater. Chem. B.

[135] Patel S., Bajpai J., Saini R., Bajpai A.K., Acharya S. (2018). Sustained release of pesticide (Cypermethrin) from nanocarriers: an effective technique for environmental and crop protection. Process Saf. Environ. Protect..

[136] de Oliveira J.L., Campos E.V.R., Pereira A.E.S., Nunes L.E.S., da Silva C.C.L., Pasquoto T., Lima R., Smaniotto G. (2018). Geraniol encapsulated in chitosan/gum Arabic nanoparticles: a promising system for pest management in sustainable agriculture. J. Agric. Food Chem..

[137] Xiao D., Liang W., Xie Z., Cheng J., Du Y., Zhao J. (2021). A temperature-responsive release cellulose-based microcapsule loaded with chlorpyrifos for sustainable pest control. J. Hazard Mater..

[138] Chi Y., Zhang G., Xiang Y., Cai D., Wu Z. (2017). Fabrication of a temperature-controlled-release herbicide using a nanocomposite. ACS Sustainable Chem. Eng..

[139] Wei K., Zhao K., Gao Y., Zhang H., Yu X., Li M.-H., Hu J. (2023). Near-infrared-light responsive degradable polysulfide pesticide carriers by one-pot inverse vulcanization. Chem. Eng. J..

[140] Sharma S., Singh S., Ganguli A.K., Shanmugam V. (2017). Anti-drift nano-stickers made of graphene oxide for targeted pesticide delivery and crop pest control. Carbon.

[141] Liu B., Zhang J., Chen C., Wang D., Tian G., Zhang G., Cai D., Wu Z. (2021). Infrared-light-responsive controlled-release pesticide using hollow carbon microspheres@polyethylene glycol/α-cyclodextrin gel. J. Agric. Food Chem..

[142] Wu W., Wan M., Fei Q., Tian Y., Song S., Shen H., Shen J. (2021). PDA@Ti_3_C_2_T_*x*_ as a novel carrier for pesticide delivery and its application in plant protection: NIR-responsive controlled release and sustained antipest activity. Pest Manag. Sci..

[143] Chen C., Zhang G., Dai Z., Xiang Y., Liu B., Bian P., Zheng K., Wu Z. (2018). Fabrication of light-responsively controlled-release herbicide using a nanocomposite. Chem. Eng. J..

[144] Gao C., Huang Q., Lan Q., Feng Y., Tang F., Hoi M.P.M., Zhang J., Lee S.M.Y. (2018). A user-friendly herbicide derived from photo-responsive supramolecular vesicles. Nat. Commun..

[145] Ye Z., Guo J., Wu D., Tan M., Xiong X., Yin Y., He G. (2015). Photo-responsive shell cross-linked micelles based on carboxymethyl chitosan and their application in controlled release of pesticide. Carbohydr. Polym..

[146] Ding K.K., Shi L.Y., Zhang L., Zeng T., Yin Y.H., Yi Y. (2016). Synthesis of photoresponsive polymeric propesticide micelles based on PEG for the controlled release of a herbicide. Polym. Chem..

[147] Fu W., Du K., Xu Z., Cheng J., Li Z., Shao X. (2023). Dual photo-controlled release system for fipronil and dinotefuran. Photochem. Photobiol. Sci..

[148] Liang Y., Gao Y., Wang W., Dong H., Tang R., Yang J., Niu J., Zhou Z. (2020). Fabrication of smart stimuli-responsive mesoporous organosilica nano-vehicles for targeted pesticide delivery. J. Hazard Mater..

[149] Ding Y., Xiao Z., Chen F., Yue L., Wang C., Fan N., Ji H., Wang Z. (2023). A mesoporous silica nanocarrier pesticide delivery system for loading acetamiprid: effectively manage aphids and reduce plant pesticide residue. Sci. Total Environ..

[150] Liang Y., Wang S., Jia H., Yao Y., Song J., Dong H., Cao Y., Zhu F., Huo Z. (2022). Pectin functionalized metal-organic frameworks as dual-stimuli-responsive carriers to improve the pesticide targeting and reduce environmental risks. Colloids Surf., B.

[151] Xu T., Wang Y., Aytac Z., Zuverza-Mena N., Zhao Z., Hu X., Ng K.W., White J.C. (2022). Enhancing agrichemical delivery and plant development with biopolymer-based stimuli responsive core-shell nanostructures. ACS Nano.

[152] Kaziem A.E., Gao Y., Zhang Y., Qin X., Xiao Y., Zhang Y., You H., Li J. (2018). α-Amylase triggered carriers based on cyclodextrin anchored hollow mesoporous silica for enhancing insecticidal activity of avermectin against *Plutella xylostella*. J. Hazard Mater..

[153] Gao Y., Liu Y., Qin X., Guo Z., Li D., Li C., Wan H., Zhu F. (2021). Dual stimuli-responsive fungicide carrier based on hollow mesoporous silica/hydroxypropyl cellulose hybrid nanoparticles. J. Hazard Mater..

[154] Fischer J., Beckers S.J., Yiamsawas D., Thines E., Landfester K., Wurm F.R. (2019). Targeted drug delivery in plants: enzyme-responsive lignin nanocarriers for the curative treatment of the worldwide grapevine trunk disease esca. Adv. Sci..

[155] Zhang D.X., Wang R., Ren C., Wang Y., Li B.X., Mu W., Liu F., Hou Y. (2022). One-step construct responsive lignin/polysaccharide/Fe nano loading system driven by digestive enzymes of lepidopteran for precise delivery of pesticides. ACS Appl. Mater. Interfaces.

[156] Liang Y., Fan C., Dong H., Zhang W., Tang G., Yang J., Jiang N., Cao Y. (2018). Preparation of MSNs-chitosan@prochloraz nanoparticles for reducing toxicity and improving release properties of prochloraz. ACS Sustainable Chem. Eng..

[157] Abdelrahman T.M., Qin X., Li D., Senosy I.A., Mmby M., Wan H., Li J., He S. (2021). Pectinase-responsive carriers based on mesoporous silica nanoparticles for improving the translocation and fungicidal activity of prochloraz in rice plants. Chem. Eng. J..

[158] Du M., Yi Y., Yin Y., Cai Z., Cai W., Li J., He G., Zhang J. (2023). Bacteria-triggered photodynamic nano-system based on hematoporphyrin-modified chitosan for sustainable plant disease control. Eur. Polym. J..

[159] Wang A., Li N., Shen Y., Sun C., Wang C., Zhao X., Cui B., Wang C. (2022). Synthesis and characterization of a novel stimuli-responsive zein nano delivery system for the controlled release of emamectin benzoate. Environ. Sci.: Nano.

[160] Monteiro R.A., Camara M.C., de Oliveira J.L., Campos E.V.R., Carvalho L.B., Proença P.L.d.F., Guilger-Casagrande M., Lima R. (2021). Zein based-nanoparticles loaded botanical pesticides in pest control: an enzyme stimuli-responsive approach aiming sustainable agriculture. J. Hazard Mater..

[161] Ernst J.W., Massey H.F. (1960). The effects of several factors on volatilization of ammonia formed from urea in the soil. Soil Sci. Soc. Am. J..

[162] Memarizadeh N., Ghadamyari M., Adeli M., Talebi K. (2014). Preparation, characterization and efficiency of nanoencapsulated imidacloprid under laboratory conditions. Ecotoxicol. Environ. Saf..

[163] Gil E.S., Hudson S.M. (2004). Stimuli-reponsive polymers and their bioconjugates. Prog. Polym. Sci..

[164] Chen N., Dempere L.A., Tong Z. (2016). Synthesis of pH-responsive lignin-based nanocapsules for controlled release of hydrophobic molecules. ACS Sustainable Chem. Eng..

[165] Xing C., Shi Z., Tian J., Sun J., Li Z. (2018). Charge-determined LCST/UCST behavior in ionic polypeptoids. Biomacromolecules.

[166] Roth P.J., Davis T.P., Lowe A.B. (2012). Comparison between the LCST and UCST transitions of double thermoresponsive diblock copolymers: insights into the behavior of POEGMA in alcohols. Macromolecules.

[167] Zhang Y., Chen W., Jing M., Liu S., Feng J., Wu H., Zhou Y., Zhang X. (2019). Self-assembled mixed micelle loaded with natural pyrethrins as an intelligent nano-insecticide with a novel temperature-responsive release mode. Chem. Eng. J..

[168] Shimoda M., Honda K. (2013). Insect reactions to light and its applications to pest management. Appl. Entomol. Zool..

[169] Huang G., Qian G., Yan Y., Xu D., Xu C., Fu L., Lin B. (2020). A super long-acting and anti-photolysis pesticide release platform through self-assembled natural polymer-based polyelectrolyte. Toxicol. Lett..

[170] Karunarathna M.H.J.S., Hatten Z.R., Bailey K.M., Lewis E.T., Morris A.L., Kolk A.R., Laib J.C., Tembo N. (2019). Reclaiming phosphate from waste solutions with Fe(III)-polysaccharide hydrogel beads for photo-controlled-release fertilizer. J. Agric. Food Chem..

[171] Li Y., Pan Y., Li B., Wang L., Xiao H. (2020). Dual-functional redox-responsive nanocarriers for loading phytohormone and complexation with heavy metal ions. J. Agric. Food Chem..

[172] Zhang S.G., Yang Y.C., Tong Z.H., Gao B., Gao N., Shen T.L., Wan Y.S., Yu Z. (2020). Self-assembly of hydrophobic and self-healing bionanocomposite-coated controlled-release fertilizers. ACS Appl. Mater. Interfaces.

[173] Zhang T., Lu Z., Wang J., Shen J., Hao Q., Li Y., Yang J., Niu Y. (2021). Preparation of mesoporous silica nanoparticle with tunable pore diameters for encapsulating and slowly releasing eugenol. Chin. Chem. Lett..

[174] Xie D., Zhao Q., Zeng X., Ma S., Zhong B., Chen Y., Zhang Q., Jia Z. (2020). Electrostatic wrapping of eupatorium-based botanical herbicide with chitosan derivatives for controlled release. Carbohydr. Polym..

[175] Aljafree N.F.A., Kamari A. (2018). Synthesis, characterisation and potential application of deoxycholic acid carboxymethyl chitosan as a carrier agent for rotenone. J. Polym. Res..

[176] Grillo R., Mattos B.D., Antunes D.R., Forini M.M.L., Monikh F.A., Rojas O.J. (2021). Foliage adhesion and interactions with particulate delivery systems for plant nanobionics and intelligent agriculture. Nano Today.

[177] Liu B., Wang Y., Yang F., Cui H., Wu D. (2018). Development of a chlorantraniliprole microcapsule formulation with a high loading content and controlled-release property. J. Agric. Food Chem..

[178] Song R., Wu Y., Bao Z., Gao Y., Zhao K., Zhang S., Zhang C., Du F. (2022). Machine learning to predict the interfacial behavior of pesticide droplets on hydrophobic surfaces for minimizing environmental risk. CS Sustainable Chem. Eng..

[179] Janprasit J., Schulte A., Crespy D. (2023). Real-time monitoring of the release of multiple payloads from nanomaterials. Chem. Commun..

[180] Stuart M.A.C., Huck W.T.S., Genzer J., Müller M., Ober C., Stamm M., Sukhorukov G.B., Szleifer I. (2010). Emerging applications of stimuli-responsive polyme. Nat. Mater..

[181] Lu Y., Sun W., Gu Z. (2014). Stimuli-responsive nanomaterials for therapeutic protein delivery. J. Contr. Release.

[182] Tian B., Liu Y., Liu J. (2021). Smart stimuli-responsive drug delivery systems based on cyclodextrin: a review. Carbohydr. Polym..

[183] Zhang Q., Kuang G., Li W., Wang J., Ren H., Zhao Y. (2023). Stimuli-responsive gene delivery nanocarriers for cancer therapy. Nano-Micro Lett..

[184] Mitter N., Hussey K. (2019). Moving policy and regulation forward for nanotechnology applications in agriculture. Nat. Nanotechnol..

[185] Xu Z., Tang T., Lin Q., Yu J., Zhang C., Zhao X., Kah M., Li L. (2022). Environmental risks and the potential benefits of nanopesticides: a review. Environ. Chem. Lett..

[186] Ramadhan Makame K., Sherif M., Ostlundh L., Sandor J., Adam B., Nagy K. (2023). Are encapsulated pesticides less harmful to human health than their conventional alternatives? Protocol for a systematic review of *in vitro* and *in vivo* animal model studies. Environ. Int..

[187] Ma Z., Gao Y., Chu F., Tong Y., He Y., Li Y., Gao Z., Chen W. (2022). Tip-assisted ambient electric arc ionization mass spectrometry for rapid detection of trace organophosphorus pesticides in strawberry. Chin. Chem. Lett..

[188] Gomes S.I.L., Scott-Fordsmand J.J., Campos E.V.R., Grillo R., Fraceto L.F., Amorim M.J.B. (2019). On the safety of nanoformulations to non-target soil invertebrates – an atrazine case study. Environ. Sci.: Nano.

[189] Blewett T.A., Qi A.A., Zhang Y., Weinrauch A.M., Blair S.D., Folkerts E.J., Sheedy C., Nilsson D. (2019). Toxicity of nanoencapsulated bifenthrin to rainbow trout (*Oncorhynchus mykiss*). Environ. Sci.: Nano.

[190] Li X., He F., Wang Z., Xing B. (2022). Roadmap of environmental health research on emerging contaminants: inspiration from the studies on engineered nanomaterials. Eco Environ. Health.

[191] Ma C., White J.C., Zhao J., Zhao Q., Xing B. (2018). Uptake of engineered nanoparticles by food crops: characterization, mechanisms, and implications. Annu. Rev. Food Sci. Technol..

[192] Su Y., Ashworth V., Kim C., Adeleye A.S., Rolshausen P., Roper C., White J., Jassby D. (2019). Delivery, uptake, fate, and transport of engineered nanoparticles in plants: a critical review and data analysis. Environ. Sci.: Nano.

[193] Zhou Q., Hu X. (2017). Systemic stress and recovery patterns of rice roots in response to graphene oxide nanosheets. Environ. Sci. Technol..

[194] Kah M., Walch H., Hofmann T. (2018). Environmental fate of nanopesticides: durability, sorption and photodegradation of nanoformulated clothianidin. Environ. Sci.: Nano.

