# Supplementary material for: Recent advances in stimuli-response mechanisms of nano-enabled controlled-release fertilizers and pesticides
Source: Eco Environ Health. 2023 Jul 23;2(3):161–75. doi: 10.1016/j.eehl.2023.07.005 (PMC10702921; doi:10.1016/j.eehl.2023.07.005)
Supplement: Multimedia component 1 [file mmc1.docx]

**Supplementary Material for**

Recent advances in stimuli-response mechanisms of nano-enabled controlled-release fertilizers and pesticides

Meimei Shen ^a^, Songlin Liu ^a^, Chuanjia Jiang ^a,*^, Tong Zhang ^a^ and Wei Chen ^a^

^a^ College of Environmental Science and Engineering, Ministry of Education Key Laboratory of Pollution Processes and Environmental Criteria, Tianjin Key Laboratory of Environmental Remediation and Pollution Control, Nankai University, Tianjin 300350, China

* Corresponding author: Chuanjia Jiang (E-mail: jiangcj@nankai.edu.cn)

**Table S1** Composition and release behaviors of stimuli-responsive controlled-release fertilizers with organic nanocarriers

| Nutrient | Carrier composition | Test medium | Response | Pot experiment | Ref. |
| --- | --- | --- | --- | --- | --- |
| Ammonium zinc phosphate | Polydopamine-poly(*N*,*N*-dimethylaminoethyl methacrylate) | Water (at pH 4.0, 7.0, and 10.0) | pH  Temp. (25, 40 ℃) | N/A | Feng et al. (2015) [1] |
| Ammonium zinc phosphate | Sodium lignosulfonate-chitosan-polydopamine | Water (at pH 4.0, 7.0, and 10.0) | pH | Effects on seedling growth of corn (*Zea mays* L.) | Li et al. (2019) [2] |
| Ammonium zinc phosphate | Zeolitic imidazolate framework-8 | Water (at pH 4.0, 7.0, and 10.0) | pH | Effects on corn growth | Ma et al. (2021) [3] |
| Ammonium nitrate | Aminated-cellulose nanofiber/ poly(acrylamide-co-2-aminoethyl methacrylate) | PBS (pH 5.5, and 7.4) and soil | pH | Effects on the N nutrition status; cell ultrastructure, cell cycle progression, and eﬃciency indicators of rice | Shaghaleh et al. (2022) [4] |
| MiZax-3 | Zeolitic imidazolate framework-8 | PBS (at pH 6.0 and 7.0) | pH | Effects on the tomato (*var. MicroTom*), pearl millet (*var. Kenya, P10*), and capsicum (*Capsicum annum*) seedlings growth | Aguliar Perez et al. (2023) [5] |
| NPK + Copper(II) | Cellulose acetate/chitosan/zein/ starch/polycaprolactone | Buffer solution (citric acid/sodium hydroxide, pH = 5) and DI water (pH 7) | pH  Enzyme (protease and α-amylase) | Effects on the seedling development and health of soybean and wheat | Xu et al. (2022) [6] |
| Urea | Carboxymethyl cellulose/poly(*N*-vinylcaprolactam-*co*-acrylamide) | Water (DI water) and soil | Temp. (10, 25, and 37 °C) | N/A | Shang et al. (2023) [7] |

Notes: N/A: not available. PBS: phosphate buffer solution. Temp.: temperature.

**Table S2** Composition and release behaviors of stimuli-responsive controlled-release fertilizers with organic–inorganic composite nanocarriers

| Nutrient | Carrier composition | Test medium | Response | Pot experiment | Ref. |
| --- | --- | --- | --- | --- | --- |
| Boron (borate) | Alginate-Mg–Al LDH | Water (buffer solutions) and soil at pH 6.5 and 7.5 | pH | Uptake of boron by *Helianthus annuus* and *Gossypium vitifolium* | Castro et al. (2020) [8] |
| Iron(Ⅱ) and NH_4_^+^ | Amino silicon oil-ethylene oxide/propylene oxide block copolymer-ferroferric oxide-palygorskite | Water (DI water) and soil | Temp. (15, 25, 35 and 45 °C) | Effects on the height, root length, chlorophyll and iron content of corn | Chi et al. (2018) [9] |
| Iron(II) | Carboxyl cellulose-attapulgite | Water (at pH 4.0, 5.0, 6.0, 7.0 and 8.0) | pH | Effects on leaf height and chlorophyll content of corn | Wang et al. (2016) [10] |
| NPK | Sodium alginate-*g*-poly(acrylic acid-*co*-acrylamide)-clinoptilolite | Water (at pH 2.0-12.0) | pH | N/A | Rashidzadeh et al. (2014) [11] |
| NPK | Sodium alginate-*g*-poly(acrylic acid-*co*-acrylamide) -montmorillonite | Water (at pH 2.0-12.0) and soil | pH | N/A | Rashidzadeh and Olad (2014) [12] |
| NPK | Maize bran-*g*-poly(acrylic acid-*co*-acrylamide)-montmorillonite | Water (at pH 2-12) and soil | pH | N/A | Olad et al. (2018) [13] |
| NPK | Salep-*g*-poly(acrylic acid)/montmorillonite | Water (at pH 2.0 and 8.0) and soil | pH | N/A | Olad et al. (2018) [14] |
| NPK | Sulfonated-carboxymethyl cellulose-*g*-poly(acrylic acid)-polyvinylpyrrolidone-silica nanoparticle | Water (at pH 2 and 8) | pH | N/A | Olad et al. (2018) [15] |
| Nitrogen and phosphorous | Banana peel cellulose-*g*-poly(acrylic acid)-polyvinyl alcohol-Mg-Al LDH | Water (salt solutions at pH 2-12) | pH | N/A | Lohmousavi et al. (2020) [16] |
| Phosphate | γ-FeOOH@biochar | DI water (at pH 1.0-11.0) and soil | pH | N/A | Wang et al. (2022) [17] |
| Selenium (Selenate) | Polyethylenimine modified hollow/mesoporous carbon nanoparticles | Water (at pH 7.4, 8.5, 10 and 11) and soil | pH  Anion | Effects on Se utilization and yield of vegetables | Zhang et al. (2018) [18] |
| Urea | Polyacrylamide/calcic montmorillonite | Water (at pH 4.0, 7.0 and 9.0) | pH | N/A | Bortolin et al. (2013) [19] |
| Urea | Cotton stalk-*g*-poly(acrylic acid)-polyvinylpyrrolidone-bentonite | Water (at pH 2-12) and soil | pH | Effects on the seed germination and growth of cotton | Wen et al. (2016) [20] |

Notes: DI water: deionized water or distilled water. N/A: not available. Temp.: temperature.

**Table S3** Composition and release behaviors of stimuli-responsive controlled-release pesticides with organic nanocarriers

| Active ingredient | Function | Carrier composition | | | Test medium | | Response | | Biocidal activity test/Pot experiment | | Ref. | |
| --- | --- | --- | --- | --- | --- | --- | --- | --- | --- | --- | --- | --- |
| Crystal violet | Antimicrobial agent | Poly(acrylic acid)-*b-*poly(*N*-isopropyl acrylamide) | | | PBS at pH 4.5, 6.0 and 7.5 | | pH  Temp. (20 and 40 ℃) | | Foliar uptake and transport in tomato (*Solanum lycopersicum*) plants | Zhang et al. (2020) [21] | |  |
| Crystal violet | Antimicrobial agent | Poly[2-(2-bromoisobutyryloxy)-ethyl methacrylate-*g*-poly(acrylic acid)-*b*-poly(*N*-isopropyl acrylamide)] | | | PBS at pH 4.5 and 7.5 | | pH  Temp. (20 and 40 ℃) | | Foliar uptake and transport in tomato (*Solanum lycopersicum*) plants | Zhang et al. (2023) [22] | |  |
| Hematoporphyrin | Antimicrobial agent | *N*-Succinyl chitosan | | | PBS at pH 7.4 | | Enzyme (esterase) | | Antibacterial activity against *Escherichia coli* and *Staphylococcus aureus*; safety on cucumber | Du et al. (2023) [23] | |  |
| Dazomet | Fungicide | Zeolitic imidazolate framework-8 | | | PBS at pH 4.8, 7.2 and 8.5 | | pH | | Fungicidal activity against *Botrytis cinerea*; toxicity to cucumber and zebrafish | Ren et al. (2022) [24] | |  |
| Diniconazole | Fungicide | Polydopamine@NH_2_-MIL-101(Fe) | | | PBS, ethanol and Tween-80 emulsifier (70:29.5:0.5, v/v/v) at pH 3.1, 7.0 and 10.3 | | pH | | Fungicidal effect against *Fusarium graminearum* | Shan et al. (2020) [25] | |  |
| Fludioxonil | Fungicide | Glycine methyl ester-conjugated polysuccinimide nanoparticles | | | PBS (pH 5.0 and 8.0, containing 30% (v/v) ethanol) and soil | | pH | | Fungicidal activity  against *Fusarium oxysporum* f. sp. *Cubense*; translocation and distribution in banana plants (*Musa* spp.) | | Wu et al. (2021) [26] | |
| Hexaconazole | Fungicide | Chitosan-tripolyphosphate | | | PBS (pH 4, 7 and 10) and soil | | pH | | Fungicidal activity against *Rhizoctonia solani* and toxicity to non-target organism (vero cells) | | Chauhan et al. (2017) [27] | |
| Penconazole | Fungicide | Chitosan/carboxymethyl chitosan | | | DI water (at pH 5.0, 5.3, 5.7, 7.0, and 9.0) | | pH | | Fungicidal activity against *Coleosporium plumeria* | | Zhou et al. (2022) [28] | |
| Prochloraz | Fungicide | | 2,4-Dinitrobenzaldehyde@ZIF-8 | PBS (pH 5.0, 6.5, and 8.0) and soil | | pH  Light (UV) | | Fungicidal activity against *Sclerotinia sclerotiorum*; uptake and translocation of in oilseed rape (*Brassica napus* L.) and the fungus | | | Liang et al. (2021) [29] | |
| Pyraclostrobin | Fungicide | Methacrylated kraft lignin | | | Water | | Enzyme (lignin-degrading enzymes) | | Fungicidal activity against *Phaeomoniella chlamydospora* and *Phaeoacremonium minimum* | | Fischer et al. (2019) [30] | |
| Pyraclostrobin | Fungicide | Iron (III)-based MOF-pectin | | | Water (DI water) | | Redox  Enzyme (pectinase) | | Fungicidal effect against *Magnaporthe oryzae*; safety on rice seedlings and toxicity to zebrafish | Liang et al. (2022) [31] | |  |
| Pyraclostrobin | Fungicide | UiO-66@hydroxypropyl cellulose | | | Buffer solution (methanol/water, 80:20, v/v) at different pH values ( 4.5, 6.0, and 7.4) | | pH  Enzyme (cellulase) | | Fungicidal activity against *Rhizoctonia solani*, acute toxicity toward *Daphnia magna*; and toxicity to rice seeds, soil microorganisms, and human liver cell | Ma et al. (2023) [32] | |  |
| Tebuconazole | Fungicide | MIL-101(Fe)/ tannic acid | | | Aqueous media containing ethanol (v_etha nol_/v_water_ = 1:4) and 0.1% Tween-80 at pH 5.0 and 9.0 in the presence of 0.5 mM EDTA or 0.5 mM PO_4_^3–^ | | pH  Light (NIR)  Redox | | Fungicidal activity against *Rhizoctonia solani* and *Fusarium graminearum* and control efficacy of wheat powdery mildew; safety on wheat | | Dong et al. (2021) [33] | |
| Tebuconazole | Microbicide (Fungicide and bactericide) | Porous porphyrinic MOFs@pectin@chitosan | | | PBS (pH 5.0 and 7.0, with 10% methanol) | | pH  Enzyme (pectinase) | | Microbicidal activity against *Xanthomonas campestris* pv. *campestris*, *Pseudomonas syringae* pv. *Tomato* and *Alternaria alternate*, and safety on Chinese cabbage | | Tang et al. (2019) [34] | |
| Diuron | Herbicide | 2-Nitrobenzyl succinate-carboxymethyl chitosan | | | PBS | | Light (UV) | | N/A | | Ye et al. (2015) [35] | |
| Diuron | Herbicide | Subabul stem lignin | | | Water (buffer at pH 5.0, 7.0 and 9.0) | | pH | | Effects on the growth of canola (*Brassica rapa*) seedlings | | Yearla and Padmasree (2016) [36] | |
| 2,4-Dichlorophenoxy acetic acid | Herbicide | Polyethyleneimine-grafted lignin microspheres | | | Water (at pH 2.0, 7.0, and 11.0) at 30 °C | | pH | | N/A | | Wu et al. (2021) [37] | |
| 2,4-Dichlorophenoxy acetic acid | Herbicide | Methoxypolyethylene glycol-*o*-nitrobenzyl | | | DI water and PBS solution (at pH 5.0, 7.0, and 9.0) | | pH  Light | | Herbicidal activity against *Arabidopsis thaliana*; safety on zebrafish | | Shan et al. (2022) [38] | |
| 2,4-Dichlorophenoxy acetic acid | Herbicide | MIL-101(Fe)/ polydopamine | | | Methanol-water mixture (10:90, v/v) at pH 4.0, 7.0, and 10.0 | | pH | | Weeding effect against chicory | | Wang et al. (2022) [39] | |
| Glyphosate | Herbicide | Sodium alginate hydrogels-dopamine-modified attapulgite | | | PBS (pH 5.5, 7.0, and 8.5) and soil | | pH | | N/A | | Zha et al. (2022) [40] | |
| Glyphosate | Herbicide | Alginate-*g*-poly(*N*-isopropyl acrylamide-*co*-*N*,*N*-diethylacrylamide)/ semi-coke | | | DI water (at pH 5.0, 7.0, and 9.0) | | pH  Temp. (20, 25, and 35 ℃)  Light | | N/A | | Zheng et al. (2022) [41] | |
| 2-Methyl-4-chlorophenoxy acetic acid | Herbicide | Poly(ethylene glycol)-*ο*-nitrobenzyl | | | Water (DI water) | | Light  (UV) | | N/A | | Ding et al. (2016) [42] | |
| Paraquat | Herbicide | Cucurbit[8]uril/ azobenzene derivative | | | Water (DI water) | | Light (UV) | | Herbicidal activity against *Estuca arundinacea*; safety on human liver cell, monkey kidney cell, zebrafish, and mouse | | Gao et al. (2018) [43] | |
| Acetamiprid | Insecticide | Alginate-chitosan | | | Acetate (pH 4.0), PBS (pH 7.0 and 10.0) | | pH | | N/A | | Kumar et al. (2015) [44] | |
| Acetamiprid | Insecticide | UiO-66-NH_2_- carboxymethyl cellulose | | | 30% ethanol solution (ethanol/water, v/v) at pH 5.0, 7.0, and 9.0 | | pH | | Insecticidal activity against aphids; effects on the germination and growth of maize | Song et al. (2022) [45] | |  |
| Avermectin | Insecticide | Isolated soy protein/carboxymethyl chitosan | | | 40% ethanol-water solution at 40 °C | | pH | | Insecticidal activity against *Plutella xylostella* | | Chen et al. (2019) [46] | |
| Avermectin | Insecticide | Soybean protein isolate-carboxymethyl cellulose | | | 40% ethanol-water solution at pH 5.0, 7.0 and 9.0 | | pH | | Insecticidal activity against diamondback moth | | Liu et al. (2019) [47] | |
| Avermectin | Insecticide | Carboxymethyl chitosan-allyl glycidyl ether-trisiloxane surfactant | | | 40% aqueous ethanol at pH 3.0, 5.0, 7.0 and 9.0 | | pH | | Insecticidal activity against *Plutella xylostella* | | Zhao et al. (2020) [48] | |
| Avermectin | Insecticide | Phosphorylated zein-carboxymethyl cellulose-*g*- poly(diallyldimethylammonium chloride) | | | 40 wt % ethanol aqueous solution at pH 3, 5, 7 and 9 | | pH | | Insecticidal activity against diamondback moth | | Hao et al. (2020) [49] | |
| Avermectin | Insecticide | Bovine serum albumin nanoparticles | | | PBS (pH 5.0, 7.0 and 9.0) | | pH  Temp. (15, 25 and 35 ℃) | | The stomach toxicity and contact toxicity of the nanoparticles to the third-instar larvae of *Monochamus alternatus* | | Su et al. (2020) [50] | |
| Avermectin | Insecticide | Polydopamine-isocyanatopropyltriethoxysilane-polyethyleneimine | | | Ethanol/water (40: 60, v/v) at pH 3.0, 7.0 and 10.0 | | pH  Enzyme (urease) | | Insecticidal activity against diamondback moth | | Wen et al. (2020) [51] | |
| Avermectin | Insecticide | 3,4-Dihydroxyhydrocinnamic acid *N*-hydroxysuccinimide ester modified chitosan | | | Buffer solutions (pH 4.5, 5.5, 6.5, 7.5 and 8.5) and methanol (1:1, v/v) mixtures | | pH | | Insecticidal activity against aphids | | Chen et al. (2021) [52] | |
| Avermectin | Insecticide | Chitosan-SS-zein | | | 40% ethanol (ethanol/water, v/v) at pH 3.0, 7.0 and 9.0 | | pH  Redox | | Insecticidal activity against *Plutella xylostella* | | Zhao et al. (2022) [53] | |
| Avermectin | Insecticide | Poly(propylene oxide-*co*-carbon dioxide-*co*-allyl glycidyl ether)-poly(*N*-isopropylacrylamide)-polyethylene glycol monomethyl ether | | | 30% ethanol aqueous solution | | Temp. (25 and 38 ℃) | | N/A | | Xu et al. (2023) [54] | |
| Avermectin | Insecticide | Chitosan-sodium lignosulfonate | | | Aqueous media containing ethanol (*V*_etha nol_/*V*_water_ = 80:20) | | pH  Enzyme (laccase) | | Insecticidal activity against *Plutella xylostella* | | Yu et al. (2023) [55] | |
| Avermectin | Insecticide | Chitosan-sodium lignosulfonate@alkaline lignin-based pickering emulsion | | | Aqueous media containing ethanol (*V*_etha_ *nol/V*_wate_*_r_* = 80:20) | | pH  Enzyme (laccase) | | Insecticidal activity against *Plutella xylostella*; safety on *Brachydanio rerio* | | Yu et al. (2023) [56] | |
| Chlorantraniliprole | Insecticide | MIL-101(Fe)- carboxymethyl starch | | | Methanol and Tris-HCl buffer mixture (30:70, v/v, pH = 6.0, 7.0, and 10.0) | | pH  Redox  Enzyme (α-amylase) | | Insecticidal activity against *Spodoptera frugiperda*; safety on maize seedlings | Liang et al. (2022) [57] | |  |
| Chlorpyrifos | Insecticide | n-hexadecane/ nanofibrillated cellulose | | | 45% ethanol water solution (ethanol/water, v/v) | | Temp. (15, 25 and 35 ℃) | | Insecticidal activity against *Plutella xylostella*; toxicity to zebrafish | | Xiao et al. (2021) [58] | |
| Chlorfenapyr | Insecticide | O-carboxymethyl chitosan | | | PBS at pH 5.0, 7.0 and 10.0 containing 1% Tween 80 | | pH | | Insecticidal activity against *Spodoptera frugiperda* and toxicity to honey bees (*Apis mellifera ligustica* Spinola) | | Hou et al. (2023) [59] | |
| λ-Cyhalothrin | Insecticide | Sodium lignosulfonate/ dodecyl dimethyl benzyl ammonium chloride/Fe(III) | | | Water (DI water) at different pH (5.0, 7.0, and 9.0) | | pH  Enzyme (laccase) | | Insecticidal activity against *Agrotis ipsilon*; toxicity to *Brachydanio rerio* | | Zhang et al. (2021) [60] | |
| λ-Cyhalothrin | Insecticide | Isopropyl myristate@poly(*N*-isopropylacrylamide-*co*-butyl methylacrylate) | | | Methanol-water mixture (4:1, v/v) | | Temp. (25, 29, and 35 ℃) | | Insecticidal activity against *Plutella xylostella*, *Aphis gossypii* and *Pieris rapae*; safety on HepG2 and *Brachydanio rerio* | | Shen et al. (2022) [61] | |
| λ-Cyhalothrin | Insecticide | Lignin/polysaccharide/Fe(III) | | | Water (DI water) | | Enzyme (laccase and cellulase) | | Insecticidal activity against *Agrotis ipsilon*; toxicity to ladybug larvae and *Trichogramma ostriniae* | | Zhang et al. (2022) [62] | |
| Cypermethrin | Insecticide | Calcium alginate | | | Water (distilled water at pH 4.6, 7.2, 9.2 and 12) and soil | | pH  Temp. (5, 25 and 37 ℃) | | N/A | | Patel et al. (2018) [63] | |
| Dinotefuran | Insecticide | MIL-101 @carboxymethyl chitosan | | | PBS at pH 5.0, 6.0 and 7.4, and citric acid solution with different concentrations (0.002, 0.01, and 0.1 mol/L) | | pH | | Insecticidal activity against planthoppers | | Feng et al. (2020) [64] | |
| Dinotefuran | Insecticide | Zeolitic imidazolate framework-8 | | | Water (at pH 4.0, 7.0, and 10.0) | | pH | | Insecticidal activity against white grub | | Ma et al. (2021) [3] | |
| Emamectin benzoate | Insecticide | Yolk lecithin-cholesterol | | | DI water | | Temp. (20, 30, and 40 ℃) | | Insecticidal activity against *Spodoptera exigua*; toxicity to zebrafish | | Du et al. (2022) [65] | |
| Emamectin benzoate | Insecticide | Zein | | | DI water (pH 7.0 and 9.0) | | pH  Enzyme (alkaline protease) | | Insecticidal activity against *Spodoptera exigua* | | Wang et al. (2022) [66] | |
| Geraniol | Insect repellent | Chitosan-gum arabic | | | 5% (w/v) Tween 80 solution | | Temp. (20, 25 and 30 ℃) | | Biological effect on whitefly (*Bemisia tabaci*) | | de Oliverira et al. (2018) [67] | |
| Imidacloprid | Insecticide | Poly(citric acid)-poly(ethylene glycol)-poly(citric acid) | | | PBS (pH 7 and 10) | | pH | | Insecticidal efficiency against *Glyphodes pyloalis* larvae | | Memarizadeh et al. (2014) [68] | |
| Imidacloprid | Insecticide | Polydopamine-poly(*N*-isopropyl acrylamide) | | | Water (DI water) | | Light (NIR)  Temp. (15, 25 and 40 ℃) | | N/A | | Xu et al. (2017) [69] | |
| Imidacloprid | Insecticide | Poly(*β*-cyclodextrin)-adamantane-grafted poly(acrylic acid) | | | Methanol at pH 5.0, 6.8, and 10.0 | | pH  Temp. (25, 35, and 45 ℃)  Enzyme (amyloglucosidase) | | Insecticidal activity against *Megoura japonica Matsumura*; toxicity to earthworms | | Wu et al. (2022) [70] | |
| Limonene and carvacrol | Insecticide | zein | | | 0.2 mol L^−1^ Tris HCl buffer at 37 °C | | Enzyme (trypsin) | | Insecticidal activity against *Spodoptera frugiperda* | | Monteiro et al. (2021) [71] | |
| Spinosad | Insecticide | Chitosan | | | Buffer solutions at pH (1.2, 4.0, 6.8, 7.4 and 9.0) | | pH  Temp. (20, 30, 37, 45 and 50 ℃) | | N/A | | Li et al. (2020) [72] | |
| Thiacloprid | Insecticide | MIL-101(Fe)/ polydopamine | | | Methanol-water mixture (10:90, v/v) at pH 4.0, 7.0, and 10.0 | | pH | | Insecticidal activity against locust | | Wang et al. (2022) [39] | |
| Thiacloprid | Insecticide | α-Cyclodextrin/ZIF-8 | | | Methanol-water mixture (30:70, v/v) at pH 5.0, 7.0, and 9.0 | | pH  Enzyme (α-amylase) | | Insecticidal activity against *Acyrthosiphon pisum*; safety on pea seeds and earthworms | | Zhang et al. (2023) [73] | |
| Salicylic acid | Phytohormone | Carboxymethyl cellulose/3,3’-dithiobis(propionohydrazide) | | | HCl solution (at pH 3.5, 5.5, and 7.0) | | Redox  pH | | N/A | Hou et al. (2019) [74] | |  |
| Spermidine | Plant stress-regulating agent | Poly[2-(2-bromoisobutyryloxy)-ethyl methacrylate-*g*-poly(acrylic acid)-*b*-poly(N-isopropyl acrylamide)] | | | Acetate buffer at pH 4.5 and 7.5 | | pH  Temp. (20 and 40 ℃) | | Effect on the photosynthesis of tomato (*Solanum lycopersicum*) under heat and light stress | Zhang et al. (2023) [22] | |  |

Notes: DI water: deionized water. N/A: not available. NIR: Near-infrared. PBS: phosphate buffer (saline) solution. Temp.: temperature.

**Table S4** Composition and release behaviors of stimuli-responsive controlled-release pesticides with inorganic nanocarriers

| Active ingredient | Function | Carrier composition | Test medium | Response | Biocidal activity test/Pot experiment | Ref. |
| --- | --- | --- | --- | --- | --- | --- |
| Kasugamycin | Antimicrobial agent | Aldehyde-functionalized ZnO quantum dots | Tris buffer solution at pH 4.5, 6.0 and 7.4 | pH | Bactericidal activity against *Acidovorax citrulli* and toxicity to watermelon seedlings | Liang et al. (2019) [75] |
| 2,4-Dichlorophenoxy acetic acid | Herbicide | Trimethylammonium-functionalized mesoporous silica nanoparticles | Water (at pH 3.0, 7.0, and 10.0), NaCl aqueous solution (0.1 M with pH of 6.8), and soil | pH  Ionic strength  Temp. (20, 30 and 40 °C) | Bioactivity to *Cucumis sativus* L. and *Triticum aestivum* L. | Cao et al. (2018) [76] |
| Diquat dibromide | Herbicide | Sulfonate-functionalized mesoporous silica nanoparticles | Water at pH 3.0, and 7.0, and Na_2_SO_3_ aqueous solution (0.1 and 0.2 M), and soil | pH | Herbicidal activity against *Datura stramonium* L. | Shan et al. (2019) [77] |
| Chlorpyrifos | Insecticide | Cu_2-_*_x_*Se-rGO | PBS at pH 4.0, 7.0 and 10 | pH  Light (wavelength ≥410 nm) | Insecticidal effect against *Pieris rapae* larvae | Sharma et al. (2017) [78] |

Notes: N/A: not available. PBS: phosphate buffer solution. Temp.: temperature.

**Table S5** Composition and release behaviors of stimuli-responsive controlled-release pesticides with organic–inorganic composite nanocarriers

| Active ingredient | Function | Carrier composition | Test medium | Response | Biocidal activity test/Pot experiment | Ref. |
| --- | --- | --- | --- | --- | --- | --- |
| Berberine | Antimicrobial agent | ZnO@ZIF-8 | PBS at pH 5.0, 6.5 and 8.0 | pH | Antibacterial activity against *Ralstonia solanacearum* | Liang et al. (2022) [79] |
| Azoxystrobin | Fungicide | Carboxymethyl chitosan-amino-functionalized mesoporous silica nanoparticles | PBS, ethanol and Tween-80 emulsifier (70: 29.5: 0.5, v/v/v) at pH 5.5, 7.3 and 8.7 | pH | Fungicidal effect on *Phytophthora infestans* | Xu et al. (2018) [80] |
| Azoxystrobin | Fungicide | Mesoporous silica nanoparticles-polydopamine-Cu^2+^ | PBS, ethanol and Tween-80 emulsifier (70: 29.5: 0.5, v/v/v) at pH 5.8, 7.2 and 8.6 | pH | Fungicidal effect against *Pyricularia oryzae* | Xu et al. (2020) [81] |
| Captan | Fungicide | rGO-Cu_2–_*_x_*Se @chitosan/ poly(styrene-*alt*-maleic acid)/pluronic F127 | Buffer solutions at pH 7.2 and 5.5 | pH | Antifungal activity against *Colletotrichum capsici* | Sharma et al. (2021) [82] |
| Carbendazim | Fungicide | Trimethylammoniumpillar[5]arene-methyl orange-functionalized mesoporous selenium | PBS (pH 3, 4.5, and 7.4) | pH | Antifungal activity against *Sclerotinia sclerotiorum*; safety on rape, cucumber, Shanghai green, and HepG2 cells | Huang et al. (2023) [83] |
| Chlorothalonil | Fungicide | Mesoporous silica nanoparticles/β-glucan | Sodium acetate buffer at pH 4; KH_2_PO_4_ buffer at pH 7; Tris buffer at pH 9 | pH  Enzyme (β-glucanase) | Bioactivity against *Magnaporthe grisea*, and toxicity to *Daphnia magna* and soil microbes | Kaziem et al. (2021) [84] |
| Dimethomorph | Fungicide | Hollow mesoporous silica-SS-chitosan oligosaccharide | PBS (pH 5.0 and 7.0) and soil | pH  Redox | Fungicidal effect against *Luffa cylindrica*; toxicity to zebrafish | Yang et al. (2022) [85] |
| Hymexazol | Fungicide | GO@Polydopamine | Water at different pH (pH 5.0, 7.0, and 9.0) | pH  Light (NIR) | Bioactivity against *Fusarium oxysporum* f. sp. *cucumebrium* Owen | Tong et al. (2018) [86] |
| Prochloraz | Fungicide | Mesoporous silica nanoparticles-chitosan | PBS at pH 4.0, 5.0, 6.0 and 7.0 | pH  Enzyme (esterase) | Antifungal activity against citrus diseases | Liang et al. (2018) [87] |
| Prochloraz | Fungicide | Mesoporous silica nanoparticles-pectin | PBS containing 0.1% Tween-80 emulsifier at pH 5, 7, and 9 | Enzyme (pectinase) | Fungicidal activity against *Magnaporthe oryzae* | Abdelrahman et al. (2021) [88] |
| Prochloraz | Fungicide | Fe-doped mesoporous silica nanoparticles/tannic acid | Water (DI water) and 0.1% Tween-80 at different pH (pH 4.0, 7.0, and 10.0) | pH | Fungicidal effect against *Rhizoctonia solani*; toxicity to human bronchial epithelial cells and zebrafish; effects on the growth of wheat | Wu et al. (2022) [89] |
| Pyraclostrobin | Fungicide | Hydroxypropyl cellulose-capped hollow mesoporous silica nanoparticles | Methanol-water mixture (30:70, v/v) at pH 3.0, 5.0 and 7.0 | pH  Enzyme (cellulase) | Fungicidal activity against *Magnaporthe oryzae* | Gao et al. (2021) [90] |
| Tebuconazole | Fungicide | Carboxylated porous carbon nanoparticles@chitosan | Aqueous ethanol solution (v_ethanol_/v_water_ = 1:4) with 0.1% Tween-80 at different pH (pH 2.0, 3.0, 5.0, and 7.0) | pH  Temp. (25, 37, 42 and 50 °C) | Fungicidal effect against *Candida albicans* | Dong et al. (2021) [91] |
| Tebuconazole | Fungicide | MoS_2_-embedded mesoporous silica nanoparticles@ cyclodextrin polymer | Aqueous media containing ethanol (ethanol/water, 1:4, v/v) and 0.1% Tween-80 at pH 5.0 and 7.4 | pH  Enzyme (α-amylase)  Light (NIR) | Fungicidal effect against *Rhizoctonia solani* and *Fusarium graminearum* | Dong et al. (2021) [92] |
| Tebuconazole | Fungicide | Biochar@soybean oil-polysulfide | Methanol-water mixture (1:1, v/v) | Light (NIR) | Antifungal activity against *Rhizoctia solani*, *Gibberella zeae*, and *Sclerotinia sclerotiorum*; safety on wheat seedings and adult zebrafish | Wei et al. (2023) [93] |
| Thiophanate-methyl | Fungicide | Poly(acrylic acid)- mesoporous nanoselenium | PBS at pH 5.6 and 7.4 | pH | Antifungal activity against *Botrytis cinerea*; effects on tomato and grapes | Liu et al. (2020) [94] |
| 2,4-Dichlorophenoxyacetic acid | Herbicide | Hollow mesoporous silica@1-tetradecanol @polydopamine | DI water (pH 7.0) | Light (NIR) | Herbicidal efficacy against chicory herb and purslane;  effects on the growth of corn, toxicity to anchovies | Ji et al. (2021) [95] |
| Glyphosate | Herbicide | Poly(vinyl alcohol)-amino silicon oil-attapulgite | Water (DI water) | Temp. (25, 40 and 50 ℃) | Control efficacy on weed (*Zoysia matrella*) | Chi et al. (2017) [96] |
| Glyphosate | Herbicide | Chitosan-diatomite/Fe_3_O_4_ | Acetic acid-acetate buffer solutions at pH 5.0, 6.5 and 8.0 | pH | Control efficacy on weeds (*Cynodon dacylon* and bristlegrass) | Xiang et al. (2017) [97] |
| Glyphosate | Herbicide | Biochar-azobenzene-amino silicon oil-attapulgite | Water (DI water) | Light (UV-Vis) | Weeding effect against Bermuda weeds; effects on *Caenorhabditis elegans* embryos | Chen et al. (2018) [98] |
| Paraquat | Herbicide | Carboxylated porous carbon nanoparticles@ chitosan | Water (at pH 2.0, 3.0, 5.0, 7.0, and 9.0) containing 0.1% Tween-80 and 2% Na_2_SO_4_ | pH  Temp. (25, 37, 42, and 50 °C) | Herbicidal efficacy against outdoor *Cynodon dactylon*; cytotoxicity to human normal hepatic cells (LO-2) and mice | Dong et al. (2021) [91] |
| Quinclorac | Herbicide | γ-FeOOH@Biochar | Water (aqueous solutions at pH 1.0-11.0) | pH | Weeding effect against barnyard grass and safety on rice | Wang et al. (2022) [17] |
| Abamectin | Insecticide | Poly(glycidyl methacrylate-*co*-acrylic acid)-hollow mesoporous silica | Methanol-water mixture (30:70, v/v) at pH 5, 7 and 10 | pH | Insecticidal activity against *Cnaphalocrocis medinalis* and safety on rice | Gao et al. (2019) [99] |
| Acetamiprid | Insecticide | Mesoporous silica-didecyl disulfide | Water (DI water) with different concentrations of GSH | Redox | Control efficacy to *Aphis craccivora* Koch; effects on *Vicia faba* L. germination rate and root length | Ding et al. (2023) [100] |
| Avermectin | Insecticide | α-Cyclodextrin-phenylamine-functionalized hollow mesoporous silica | Acetone-water mixture (30:70, v/v) | Enzyme (α-amylase) | Insecticidal effects against *Plutella xylostella* | Kaziem et al. (2018) [101] |
| Avermectin | Insecticide | Starch-mesoporous silica nanoparticles | Methanol–water mixture  (30:70, v/v) | Redox  Enzyme (α-amylase) | Toxicity to *Plutella xylostella* larvae | Liang et al. (2020) [102] |
| Avermectin | Insecticide | 3-Mercaptopropyl trimethoxysilane and poly(ethylene glycol) diacrylate functionalized boron nitride nanoplatelets | 40 wt% ethanol–water solvents at pH 5, 7 and 11 | pH | Insecticidal efficiency against *Plutella xylostella* | Hao et al. (2020) [103] |
| Buprofezin | Insecticide | Mesoporous silica nanoparticles-poly (*N*-isopropyl acrylamide- methacrylic acid) | Methanol–water (20:80) solution | Temp. (25, 30 and 35 ℃) | Insecticidal efficiency against *Nilaparvata lugens* | Yang et al. (2021) [104] |
| Chlorantraniliprole | Insecticide | MIL-101(Fe)@silica | A mixture of methanol and tris-HCl buffer (pH 6.0, 7.0, or 10.0) | pH | Insecticidal effect against *Plutella xylostella*; effects on *Brassica oleracea* | Gao et al. (2021) [105] |
| Chlorantraniliprole | Insecticide | α-Cyclodextrin-hollow mesoporous silica | Acetone–water mixture (30:70, v/v) at pH 5, 7 and 10 | pH  Temp. (25, 35 and 45 ℃)  Enzyme (α-amylase) | Biological activity against *Plutella xylostella* | Kaziem et al. (2017) [106] |
| Chlorpyrifos | Insecticide | Polydopamine-attapulgite -calcium alginate | PBS (pH 5.5, 7.0 and 8.5) | pH | Control efficacy against grubs | Xiang et al. (2018) [107] |
| Chlorpyrifos | Insecticide | Halloysite nanotubes/Ca^2+^/EDTA^2-^/calcium alginate | Tween aqueous solution at pH 4.0, 5.5, 7.0 and 8.5, with coexisting anions (SO_4_^2-^, CO_3_^2–^ and Cl^-^) | pH | Insecticidal effect against corn borers; toxicity to zebrafish | Teng et al. (2023) [108] |
| *β*-Cyfluthrin | Insecticide | MXene (Ti_3_C_2_)-tannic acid | 30% ethanol solution at pH 5.0, 6.0, and 7.0 (PBS) | pH | Insecticidal activity against *Culex pipiens pallens*; toxicity to maize and *Vigna radiata* (Linn.) Wilczek | Wan et al. (2022) [109] |
| λ-Cyhalothrin | Insecticide | Alginate-grafted anisotropic silica | 25% methanol solution at pH 2.0, 3.0, 4.0, 6.2, 8.0 and 9.0 | pH | N/A | Chen et al. (2017) [110] |
| λ-Cyhalothrin | Insecticide | Poly(*N*-isopropyl acrylamide)-graphene oxide | Ethanol-water mixture (3:7, v/v) | Temp. (27, 30 and 35 ℃) | N/A | Wang et al. (2021) [111] |
| Cypermethrin | Insecticide | Chitosan-diatomite/Fe_3_O_4_ | Acetic acid-acetate buffer solutions at pH 5.0, 6.5 and 8.0 | pH | Control efficacy to corn borers and cotton bollworms | Xiang et al. (2017) [97] |
| Deltamethrin | Insecticide | Graphene oxide-carboxymethyl chitosan | 30% ethanol solution at pH 5.0, 6.0, and 7.0 (adjusted with PBS) | pH | Insecticidal activity against *Culex pipiens pallens*; toxicity to maize seeds | Song et al. (2022) [112] |
| Dinotefuran | Insecticide | Hollow mesoporous silica@1-tetradecanol @polydopamine | DI water (pH 4.0 and 7.0) | pH | Insecticidal activity against aphids and grubs; toxicity to the corn and anchovies | Ji et al. (2021) [95] |
| Dinotefuran | Insecticide | Thioacetal *o*-nitrobenzaldehyde | Aqueous methanol (5.0× 10^−5^ M) | Light (UV and sunlight) | Insecticidal activity against *Mythimna separata*, *Aphis craccivora*, and *Culex pipiens pallens* | Fu et al. (2023) [113] |
| Emamectin benzoate | Insecticide | Carboxymethyl chitosan@carbon nanoparticles | PBS (pH 5.0, 6.0 and 7.0) | pH | Control efficacy to *Mythimna separata* | Song et al. (2019) [114] |
| Emamectin benzoate | Insecticide | Polydopamine-Ti_3_C_2_T*_x_* | 30% ethanol solution | Light (NIR) | Insecticidal activity against *Pyrausta nubilalis* | Wu et al. (2021) [115] |
| Fipronil | Insecticide | Thioacetal *o*-nitrobenzaldehyde | Aqueous methanol (5.0× 10^−5^ M) | Light (UV and sunlight) | Insecticidal activity against *Mythimna separata*, *Aphis craccivora*, and *Culex pipiens pallens* | Fu et al. (2023) [113] |
| Imidacloprid | Insecticide | Hollow carbon microspheres-polyethylene glycol/α-cyclodextrin gel | Water (DI water) | Light (infrared) | Control efficacy to corn borers | Liu et al. (2021) [116] |
| Thiamethoxam | Insecticide | Poly(*N*-isopropyl acrylamide-*co*-methacrylic acid)-hollow mesoporous silica | Methanol-water mixture (30:70, v/v) | Temp. (28, 31 and 34 ℃) | Insecticidal effects against *Nilaparvata lugens*; effects on rice seedlings | Gao et al. (2020) [117] |

Notes: DI water: deionized water or distilled water. NIR: Near-infrared. N/A: not available. PBS: phosphate buffer (saline) solution. Temp.: temperature.

**REFERENCES**

[1] C. Feng, S. Lü, C. Gao, X. Wang, X. Xu, X. Bai, N. Gao, M. Liu, L. Wu, “Smart” fertilizer with temperature- and pH-responsive behavior via surface-initiated polymerization for controlled release of nutrients, ACS Sustainable Chem. Eng. 3 (2015) 3157-3166. <https://doi.org/10.1021/acssuschemeng.5b01384>.

[2] T. Li, S.Y. Lu, J. Yan, X. Bai, C.M. Gao, M.Z. Liu, An environment-friendly fertilizer prepared by layer-by-layer self-assembly for pH-responsive nutrient release, ACS Appl. Mater. Interfaces 11 (2019) 10941-10950. <https://doi.org/10.1021/acsami.9b01425>.

[3] S. Ma, Y. Ji, Y. Dong, S. Chen, Y. Wang, S. Lu, An environmental-friendly pesticide-fertilizer combination fabricated by in-situ synthesis of ZIF-8, Sci. Total Environ. 789 (2021) 147845. <https://doi.org/10.1016/j.scitotenv.2021.147845>.

[4] H. Shaghaleh, Y. Alhaj Hamoud, X. Xu, S. Wang, H. Liu, A pH-responsive/sustained release nitrogen fertilizer hydrogel based on aminated cellulose nanofiber/cationic copolymer for application in irrigated neutral soils, J. Clean. Prod. 368 (2022) 133098. <https://doi.org/10.1016/j.jclepro.2022.133098>.

[5] K.M. Aguliar Perez, Y. Alagoz, B. Maatouk, J.Y. Wang, L. Berqdar, S. Qutub, M. Jamil, S. AlNasser, N. BinSaleh, P. Lin, L. Almarwaey, T. Asami, S. Al-Babili, N.M. Khashab, Biomimetic mineralization for smart biostimulant delivery and crop micronutrients fortification, Nano Lett. 23 (2023) 4732-4740. <https://doi.org/10.1021/acs.nanolett.2c04506>.

[6] T. Xu, Y. Wang, Z. Aytac, N. Zuverza-Mena, Z. Zhao, X. Hu, K.W. Ng, J.C. White, P. Demokritou, Enhancing agrichemical delivery and plant development with biopolymer-based stimuli responsive core-shell nanostructures, ACS Nano 16 (2022) 6034-6048. <https://doi.org/10.1021/acsnano.1c11490>.

[7] H. Shang, X. Yang, H. Liu, Temperature-responsive hydrogel prepared from carboxymethyl cellulose-stabilized N-vinylcaprolactam with potential for fertilizer delivery, Carbohydr. Polym. 313 (2023) 120875. <https://doi.org/10.1016/j.carbpol.2023.120875>.

[8] G.F.d. Castro, E.M. Mattiello, J.A. Ferreira, L. Zotarelli, J. Tronto, Synthesis, characterization and agronomic use of alginate microspheres containing layered double hydroxides intercalated with borate, New J. Chem. 44 (2020) 10066-10075. <https://doi.org/10.1039/C9NJ06042A>.

[9] Y. Chi, G.L. Zhang, Y.B. Xiang, D.Q. Cai, Z.Y. Wu, Fabrication of reusable temperature-controlled-released fertilizer using a palygorskite-based magnetic nanocomposite, Appl. Clay Sci. 161 (2018) 194-202. <https://doi.org/10.1016/j.clay.2018.04.024>.

[10] M. Wang, G.L. Zhang, L.L. Zhou, D.F. Wang, N.Q. Zhong, D.Q. Cai, Z.Y. Wu, Fabrication of pH-controlled-release ferrous foliar fertilizer with high adhesion capacity based on nanobiomaterial, ACS Sustainable Chem. Eng. 4 (2016) 6800-6808. <https://doi.org/10.1021/acssuschemeng.6b01761>.

[11] A. Rashidzadeh, A. Olad, D. Salari, A. Reyhanitabar, On the preparation and swelling properties of hydrogel nanocomposite based on sodium alginate-*g*-Poly (acrylic acid-*co*-acrylamide)/Clinoptilolite and its application as slow release fertilizer, J. Polym. Res. 21 (2014) 1-15. <https://doi.org/10.1007/s10965-013-0344-9>.

[12] A. Rashidzadeh, A. Olad, Slow-released NPK fertilizer encapsulated by NaAlg-*g*-poly(AA-*co*-AAm)/MMT superabsorbent nanocomposite, Carbohydr. Polym. 114 (2014) 269-278. <https://doi.org/10.1016/j.carbpol.2014.08.010>.

[13] A. Olad, H. Gharekhani, A. Mirmohseni, A. Bybordi, Superabsorbent nanocomposite based on maize bran with integration of water-retaining and slow-release NPK fertilizer, Adv. Polym. Tech. 37 (2018) 1682-1694. <https://doi.org/10.1002/adv.21825>.

[14] A. Olad, H. Zebhi, D. Salari, A. Mirmohseni, A.R. Tabar, Water retention and slow release studies of a salep-based hydrogel nanocomposite reinforced with montmorillonite clay, New J. Chem. 42 (2018) 2758-2766. <https://doi.org/10.1039/c7nj03667a>.

[15] A. Olad, H. Zebhi, D. Salari, A. Mirmohseni, A.R. Tabar, Slow-release NPK fertilizer encapsulated by carboxymethyl cellulose-based nanocomposite with the function of water retention in soil, Mater. Sci. Eng., C 90 (2018) 333-340. <https://doi.org/10.1016/j.msec.2018.04.083>.

[16] S.M. Lohmousavi, H.H.S. Abad, G. Noormohammadi, B. Delkhosh, Synthesis and characterization of a novel controlled release nitrogen-phosphorus fertilizer hybrid nanocomposite based on banana peel cellulose and layered double hydroxides nanosheets, Arabian J. Chem. 13 (2020) 6977-6985. <https://doi.org/10.1016/j.arabjc.2020.06.042>.

[17] Y. Wang, Z. Peng, Y. Yang, Z. Li, Y. Wen, M. Liu, S. Li, L. Su, Z. Zhou, Y. Zhu, N. Zhou, Auricularia auricula biochar supported γ-FeOOH nanoarrays for electrostatic self-assembly and pH-responsive controlled release of herbicide and fertilizer, Chem. Eng. J. 437 (2022) 134984. <http://doi.org/10.1016/j.cej.2022.134984>.

[18] G. Zhang, L. Zhou, D. Cai, Z. Wu, Anion-responsive carbon nanosystem for controlling selenium fertilizer release and improving selenium utilization efficiency in vegetables, Carbon 129 (2018) 711-719. <https://doi.org/10.1016/j.carbon.2017.12.062>.

[19] A. Bortolin, F.A. Aouada, L.H.C. Mattoso, C. Ribeiro, Nanocomposite PAAm/methyl cellulose/montmorillonite hydrogel: Evidence of synergistic effects for the slow release of fertilizers, J. Agric. Food Chem. 61 (2013) 7431-7439. <https://doi.org/10.1021/jf401273n>.

[20] P. Wen, Z.S. Wu, Y.H. He, B.C. Ye, Y.J. Han, J. Wang, X.Y. Guan, Microwave-assisted synthesis of a semi-interpenetrating polymer network slow-release nitrogen fertilizer with water absorbency from cotton stalks, ACS Sustainable Chem. Eng. 4 (2016) 6572-6579. <https://doi.org/10.1021/acssuschemeng.6b01466>.

[21] Y. Zhang, J. Yan, A. Avellan, X. Gao, K. Matyjaszewski, R.D. Tilton, G.V. Lowry, Temperature- and pH-responsive star polymers as nanocarriers with potential for in vivo agrochemical delivery, ACS Nano 14 (2020) 10954-10965. <https://doi.org/10.1021/acsnano.0c03140>.

[22] Y. Zhang, L. Fu, M.R. Martinez, H. Sun, V. Nava, J. Yan, K. Ristroph, S.E. Averick, B. Marelli, J.P. Giraldo, K. Matyjaszewski, R.D. Tilton, G.V. Lowry, Temperature-responsive bottlebrush polymers deliver a stress-regulating agent in vivo for prolonged plant heat stress mitigation, ACS Sustainable Chem. Eng. 11 (2023) 3346-3358. <https://doi.org/10.1021/acssuschemeng.2c06461>.

[23] M. Du, Y. Yi, Y. Yin, Z. Cai, W. Cai, J. Li, G. He, J. Zhang, Bacteria-triggered photodynamic nano-system based on hematoporphyrin-modified chitosan for sustainable plant disease control, Eur. Polym. J. 191 (2023) 112035. <https://doi.org/10.1016/j.eurpolymj.2023.112035>.

[24] L. Ren, J. Zhao, W. Li, Q. Li, D. Zhang, W. Fang, D. Yan, Y. Li, Q. Wang, X. Jin, A. Cao, Site-specific controlled-release imidazolate framework-8 for dazomet smart delivery to improve the effective utilization rate and reduce biotoxicity, J. Agric. Food Chem. 70 (2022) 5993-6005. <https://doi.org/10.1021/acs.jafc.2c00353>.

[25] Y. Shan, C. Xu, H. Zhang, H. Chen, M. Bilal, S. Niu, L. Cao, Q. Huang, Polydopamine-modified metal–organic frameworks, NH_2_-Fe-MIL-101, as pH-sensitive nanocarriers for controlled pesticide release, Nanomaterials 10 (2020) 2000. <https://doi.org/10.3390/nano10102000>.

[26] H. Wu, P. Hu, Y. Xu, C. Xiao, Z. Chen, X. Liu, J. Jia, H. Xu, Phloem delivery of fludioxonil by plant amino acid transporter-mediated polysuccinimide nanocarriers for controlling fusarium wilt in banana, J. Agric. Food Chem. 69 (2021) 2668-2678. <https://doi.org/10.1021/acs.jafc.0c07028>.

[27] N. Chauhan, N. Dilbaghi, M. Gopal, R. Kumar, K.H. Kim, S. Kumar, Development of chitosan nanocapsules for the controlled release of hexaconazole, Int. J. Biol. Macromol. 97 (2017) 616-624. <https://doi.org/10.1016/j.ijbiomac.2016.12.059>.

[28] Y. Zhou, J. Wu, J. Zhou, S. Lin, D. Cheng, pH-responsive release and washout resistance of chitosan-based nano-pesticides for sustainable control of plumeria rust, Int. J. Biol. Macromol. 222 (2022) 188-197. <https://doi.org/10.1016/j.ijbiomac.2022.09.144>.

[29] W. Liang, Z. Xie, J. Cheng, D. Xiao, Q. Xiong, Q. Wang, J. Zhao, W. Gui, A light-triggered pH-responsive metal–organic framework for smart delivery of fungicide to control sclerotinia diseases of oilseed rape, ACS Nano 15 (2021) 6987-6997. <https://doi.org/10.1021/acsnano.0c10877>.

[30] J. Fischer, S.J. Beckers, D. Yiamsawas, E. Thines, K. Landfester, F.R. Wurm, Targeted drug delivery in plants: Enzyme-responsive lignin nanocarriers for the curative treatment of the worldwide grapevine trunk disease esca, Adv. Sci. 6 (2019) 1802315. <https://doi.org/10.1002/advs.201802315>.

[31] Y. Liang, S. Wang, H. Jia, Y. Yao, J. Song, H. Dong, Y. Cao, F. Zhu, Z. Huo, Pectin functionalized metal-organic frameworks as dual-stimuli-responsive carriers to improve the pesticide targeting and reduce environmental risks, Colloids Surf., B 219 (2022) 112796. <https://doi.org/10.1016/j.colsurfb.2022.112796>.

[32] Y. Ma, M. Yu, Y. Wang, S. Pan, X. Sun, R. Zhao, Z. Sun, R. Gao, X. Guo, Y. Xu, X. Wu, A pH/cellulase dual stimuli-responsive cellulose-coated metal–organic framework for eco-friendly fungicide delivery, Chem. Eng. J. 462 (2023) 142190. <https://doi.org/10.1016/j.cej.2023.142190>.

[33] J. Dong, W. Chen, J. Feng, X. Liu, Y. Xu, C. Wang, W. Yang, X. Du, Facile, smart, and degradable metal–organic framework nanopesticides gated with FeIII-tannic acid networks in response to seven biological and environmental stimuli, ACS Appl. Mater. Interfaces 13 (2021) 19507-19520. <https://doi.org/10.1021/acsami.1c04118>.

[34] J. Tang, G. Ding, J. Niu, W. Zhang, G. Tang, Y. Liang, C. Fan, H. Dong, J. Yang, J. Li, Y. Cao, Preparation and characterization of tebuconazole metal-organic framework-based microcapsules with dual-microbicidal activity, Chem. Eng. J. 359 (2019) 225-232. <https://doi.org/10.1016/j.cej.2018.11.147>.

[35] Z. Ye, J. Guo, D. Wu, M. Tan, X. Xiong, Y. Yin, G. He, Photo-responsive shell cross-linked micelles based on carboxymethyl chitosan and their application in controlled release of pesticide, Carbohydr. Polym. 132 (2015) 520-528. <https://doi.org/10.1016/j.carbpol.2015.06.077>.

[36] S.R. Yearla, K. Padmasree, Exploitation of subabul stem lignin as a matrix in controlled release agrochemical nanoformulations: a case study with herbicide diuron, Environ. Sci. Pollut. Res. 23 (2016) 18085-18098. <https://doi.org/10.1007/s11356-016-6983-8>.

[37] H. Wu, L. Gong, X. Zhang, F. He, Z. Li, Bifunctional porous polyethyleneimine-grafted lignin microspheres for efficient adsorption of 2,4-dichlorophenoxyacetic acid over a wide pH range and controlled release, Chem. Eng. J. 411 (2021) 128539. <https://doi.org/10.1016/j.cej.2021.128539>.

[38] P. Shan, Y. Lu, W. Lu, X. Yin, H. Liu, D. Li, X. Lian, W. Wang, Z. Li, Z. Li, Biodegradable and light-responsive polymeric nanoparticles for environmentally safe herbicide delivery, ACS Appl. Mater. Interfaces 14 (2022) 43759-43770. <https://doi.org/10.1021/acsami.2c12106>.

[39] Y. Wang, S. Ma, X. Yang, Y. Li, S. Lü, Facile synthesis of the dual pesticide-loaded metal–organic framework hybrid for pH-responsive release, ACS Agric. Sci. Technol. 2 (2022) 1267-1275. <https://doi.org/10.1021/acsagscitech.2c00222>.

[40] X. Zha, X. Hou, Q. Li, H. Nan, F. Ge, Y. Liu, F. Li, D. Zhang, J. Tian, Loading glyphosate in attapulgite and sodium alginate hydrogels to construct pH-responsive controlled release microsphere for enhanced soil sustained release, ACS Agric. Sci. Technol. 2 (2022) 1090-1100. <https://doi.org/10.1021/acsagscitech.2c00195>.

[41] D. Zheng, K. Wang, B. Bai, N. Hu, H. Wang, Swelling and glyphosate-controlled release behavior of multi-responsive alginate-g-P(NIPAm-*co*-NDEAm)-based hydrogel, Carbohydr. Polym. 282 (2022) 119113. <https://doi.org/10.1016/j.carbpol.2022.119113>.

[42] K.K. Ding, L.Y. Shi, L. Zhang, T. Zeng, Y.H. Yin, Y. Yi, Synthesis of photoresponsive polymeric propesticide micelles based on PEG for the controlled release of a herbicide, Polym. Chem. 7 (2016) 899-904. <https://doi.org/10.1039/c5py01690h>.

[43] C. Gao, Q. Huang, Q. Lan, Y. Feng, F. Tang, M.P.M. Hoi, J. Zhang, S.M.Y. Lee, R. Wang, A user-friendly herbicide derived from photo-responsive supramolecular vesicles, Nat. Commun. 9 (2018) 2967. <https://doi.org/10.1038/s41467-018-05437-5>.

[44] S. Kumar, N. Chauhan, M. Gopal, R. Kumar, N. Dilbaghi, Development and evaluation of alginate-chitosan nanocapsules for controlled release of acetamiprid, Int. J. Biol. Macromol. 81 (2015) 631-637. <https://doi.org/10.1016/j.ijbiomac.2015.08.062>.

[45] S. Song, M. Wan, W. Feng, Y. Tian, X. Jiang, Y. Luo, J. Shen, Environmentally friendly Zr-based MOF for pesticide delivery: Ultrahigh loading capacity, pH-responsive release, improved leaf affinity, and enhanced antipest activity, Langmuir 38 (2022) 10867-10874. <https://doi.org/10.1021/acs.langmuir.2c01556>.

[46] L. Chen, X. Zhou, G. Lin, H. Chen, L. Hao, H. Zhou, Synthesis of pH‐responsive isolated soy protein/carboxymethyl chitosan microspheres for sustained pesticide release, J. Appl. Polym. Sci. 137 (2019) 48358. <https://doi.org/10.1002/app.48358>.

[47] G. Liu, G. Lin, M. Tan, H. Zhou, H. Chen, H. Xu, X. Zhou, Hydrazone-linked soybean protein isolate-carboxymethyl cellulose conjugates for pH-responsive controlled release of pesticides, Polym. J. 51 (2019) 1211-1222. <https://doi.org/10.1038/s41428-019-0235-y>.

[48] M. Zhao, H. Zhou, L. Chen, L. Hao, H. Chen, X. Zhou, Carboxymethyl chitosan grafted trisiloxane surfactant nanoparticles with pH sensitivity for sustained release of pesticide, Carbohydr. Polym. 243 (2020) 116433. <https://doi.org/10.1016/j.carbpol.2020.116433>.

[49] L. Hao, G. Lin, J. Lian, L. Chen, H. Zhou, H. Chen, H. Xu, X. Zhou, Carboxymethyl cellulose capsulated zein as pesticide nano-delivery system for improving adhesion and anti-UV properties, Carbohydr. Polym. 231 (2020) 115725. <https://doi.org/10.1016/j.carbpol.2019.115725>.

[50] C. Su, Y. Ji, S. Liu, S. Gao, S. Cao, X. Xu, C. Zhou, Y. Liu, Fluorescence-labeled abamectin nanopesticide for comprehensive control of pinewood nematode and monochamus alternatus hope, ACS Sustainable Chem. Eng. 8 (2020) 16555-16564. <https://doi.org/10.1021/acssuschemeng.0c05771>.

[51] H. Wen, H. Zhou, L. Hao, H. Chen, H. Xu, X. Zhou, Enzyme cum pH dual-responsive controlled release of avermectin from functional polydopamine microcapsules, Colloids Surf., B 186 (2020) 110699. <https://doi.org/10.1016/j.colsurfb.2019.110699>.

[52] H. Chen, H. Zhi, J. Liang, M. Yu, B. Cui, X. Zhao, C. Sun, Y. Wang, H. Cui, Z. Zeng, Development of leaf-adhesive pesticide nanocapsules with pH-responsive release to enhance retention time on crop leaves and improve utilization efficiency, J. Mater. Chem. B 9 (2021) 783-792. <https://doi.org/10.1039/d0tb02430a>.

[53] M. Zhao, P. Li, H. Zhou, L. Hao, H. Chen, X. Zhou, pH/redox dual responsive from natural polymer-based nanoparticles for on-demand delivery of pesticides, Chem. Eng. J. 435 (2022) 134861. <https://doi.org/10.1016/j.cej.2022.134861>.

[54] J. Xu, H. Li, Y. Niu, Synthesis of a temperature sensitive graft carbon dioxide‐based copolymer and its evaluation as a nano drug carrier, Polym. Adv. Technol. 34 (2023) 2235-2247. <https://doi.org/10.1002/pat.6044>.

[55] X. Yu, J. Wang, X. Li, S. Ma, W. Zhu, H. Wang, Dual-responsive microcapsules with tailorable shells from oppositely charged biopolymers for precise pesticide release, Mater. Adv. 4 (2023) 1089-1100. <https://doi.org/10.1039/d2ma01046a>.

[56] X. Yu, X. Li, S. Ma, Y. Wang, W. Zhu, H. Wang, Biomass‐based, interface tunable, and dual‐responsive pickering emulsions for smart release of pesticides, Adv. Funct. Mater. (2023) 2214911. https://doi.org/10.1002/adfm.202214911.

[57] Y. Liang, S. Wang, H. Jia, Y. Yao, J. Song, W. Yang, Y. Cao, F. Zhu, Z. Huo, pH/redox/α-amylase triple responsive metal-organic framework composites for pest management and plant growth promotion, Microporous Mesoporous Mater. 344 (2022) 112230. <https://doi.org/10.1016/j.micromeso.2022.112230>.

[58] D. Xiao, W. Liang, Z. Xie, J. Cheng, Y. Du, J. Zhao, A temperature-responsive release cellulose-based microcapsule loaded with chlorpyrifos for sustainable pest control, J. Hazard. Mater. 403 (2021) 123654. <https://doi.org/10.1016/j.jhazmat.2020.123654>.

[59] R. Hou, C. Li, Y. Tan, Y. Wang, S. Huang, C. Zhao, Z. Zhang, Eco-friendly *O*-carboxymethyl chitosan base chlorfenapyr nanopesticide for effective pest control and reduced toxicity to honey bees, Int. J. Biol. Macromol. 224 (2023) 972-983. <https://doi.org/10.1016/j.ijbiomac.2022.10.182>.

[60] D.X. Zhang, J. Du, R. Wang, J. Luo, T.F. Jing, B.X. Li, W. Mu, F. Liu, Y. Hou, Core/shell dual‐responsive nanocarriers via iron‐mineralized electrostatic self‐assembly for precise pesticide delivery, Adv. Funct. Mater. 31 (2021) 2102027. <https://doi.org/10.1002/adfm.202102027>.

[61] Y. Shen, C. An, J. Jiang, B. Huang, N. Li, C. Sun, C. Wang, S. Zhan, X. Li, F. Gao, X. Zhao, H. Cui, R. Gooneratne, Y. Wang, Temperature-dependent nanogel for pesticide smart delivery with improved foliar dispersion and bioactivity for efficient control of multiple pests, ACS Nano 16 (2022) 20622-20632. <https://doi.org/10.1021/acsnano.2c07517>.

[62] D.X. Zhang, R. Wang, C. Ren, Y. Wang, B.X. Li, W. Mu, F. Liu, Y. Hou, One-step construct responsive lignin/polysaccharide/Fe nano loading system driven by digestive enzymes of lepidopteran for precise delivery of pesticides, ACS Appl. Mater. Interfaces 14 (2022) 41337-41347. <https://doi.org/10.1021/acsami.2c10899>.

[63] S. Patel, J. Bajpai, R. Saini, A.K. Bajpai, S. Acharya, Sustained release of pesticide (cypermethrin) from nanocarriers: An effective technique for environmental and crop protection, Process Saf. Environ. Prot. 117 (2018) 315-325. <https://doi.org/10.1016/j.psep.2018.05.012>.

[64] P. Feng, J. Chen, C. Fan, G. Huang, Y. Yu, J. Wu, B. Lin, An eco-friendly MIL-101@CMCS double -coated dinotefuran for long-acting active release and sustainable pest control, J. Cleaner Prod. 265 (2020) 121851. <https://doi.org/10.1016/j.jclepro.2020.121851>.

[65] Q. Du, L. Chen, X. Ding, B. Cui, H. Chen, F. Gao, Y. Wang, H. Cui, Z. Zeng, Development of emamectin benzoate-loaded liposome nano-vesicles with thermo-responsive behavior for intelligent pest control, J. Mater. Chem. B 10 (2022) 9896-9905. <https://doi.org/10.1039/D2TB02080G>.

[66] A. Wang, N. Li, Y. Shen, C. Sun, C. Wang, X. Zhao, B. Cui, C. Wang, S. Zhan, X. Li, H. Cui, Y. Wang, Synthesis and characterization of a novel stimuli-responsive zein nano delivery system for the controlled release of emamectin benzoate, Environ. Sci.: Nano 9 (2022) 4411-4422. <https://doi.org/10.1039/D2EN00720G>.

[67] J.L. de Oliveira, E.V.R. Campos, A.E.S. Pereira, L.E.S. Nunes, C.C.L. da Silva, T. Pasquoto, R. Lima, G. Smaniotto, R.A. Polanczyk, L.F. Fraceto, Geraniol encapsulated in chitosan/gum arabic nanoparticles: A promising system for pest management in sustainable agriculture, J. Agric. Food Chem. 66 (2018) 5325-5334. <https://doi.org/10.1021/acs.jafc.8b00331>.

[68] N. Memarizadeh, M. Ghadamyari, M. Adeli, K. Talebi, Preparation, characterization and efficiency of nanoencapsulated imidacloprid under laboratory conditions, Ecotoxicol. Environ. Saf. 107 (2014) 77-83. <https://doi.org/10.1016/j.ecoenv.2014.05.009>.

[69] X.H. Xu, B. Bai, H.L. Wang, Y.R. Suo, A near-infrared and temperature-responsive pesticide release platform through core-shell polydopamine@PNIPAm nanocomposites, ACS Appl. Mater. Interfaces 9 (2017) 6424-6432. <https://doi.org/10.1021/acsami.6b15393>.

[70] T. Wu, K. Zhao, C. Zhang, T. Zhong, Z. Li, Z. Bao, Y. Gao, F. Du, Promising delivery platform for smart pest control with high water-retaining capacity, ACS Appl. Mater. Interfaces 14 (2022) 55062-55074. <https://doi.org/10.1021/acsami.2c15737>.

[71] R.A. Monteiro, M.C. Camara, J.L. de Oliveira, E.V.R. Campos, L.B. Carvalho, P.L.d.F. Proença, M. Guilger-Casagrande, R. Lima, J. do Nascimento, K.C. Gonçalves, R.A. Polanczyk, L.F. Fraceto, Zein based-nanoparticles loaded botanical pesticides in pest control: An enzyme stimuli-responsive approach aiming sustainable agriculture, J. Hazard. Mater. 417 (2021) 126004. <https://doi.org/10.1016/j.jhazmat.2021.126004>.

[72] G.B. Li, J. Wang, X.P. Kong, Coprecipitation-based synchronous pesticide encapsulation with chitosan for controlled spinosad release, Carbohydr. Polym. 249 (2020) 116865. <https://doi.org/10.1016/j.carbpol.2020.116865>.

[73] X. Zhang, Y. He, Z. Yuan, G. Shen, Z. Zhang, J. Niu, L. He, J. Wang, K. Qian, A pH- and enzymatic-responsive nanopesticide to control pea aphids and reduce toxicity for earthworms, Sci. Total Environ. 861 (2023) 160610. <https://doi.org/10.1016/j.scitotenv.2022.160610>.

[74] X. Hou, Y. Pan, H. Xiao, J. Liu, Controlled release of agrochemicals using pH and redox dual-responsive cellulose nanogels, J. Agric. Food Chem. 67 (2019) 6700-6707. <https://doi.org/10.1021/acs.jafc.9b00536>.

[75] Y. Liang, Y. Duan, C. Fan, H. Dong, J. Yang, J. Tang, G. Tang, W. Wang, N. Jiang, Y. Cao, Preparation of kasugamycin conjugation based on ZnO quantum dots for improving its effective utilization, Chem. Eng. J. 361 (2019) 671-679. <https://doi.org/10.1016/j.cej.2018.12.129>.

[76] L. Cao, Z. Zhou, S. Niu, C. Cao, X. Li, Y. Shan, Q. Huang, Positive-charge functionalized mesoporous silica nanoparticles as nanocarriers for controlled 2,4-dichlorophenoxy acetic acid sodium salt release, J. Agric. Food Chem. 66 (2018) 6594-6603. <https://doi.org/10.1021/acs.jafc.7b01957>.

[77] Y. Shan, L. Cao, C. Xu, P. Zhao, C. Cao, F. Li, B. Xu, Q. Huang, Sulfonate-functionalized mesoporous silica nanoparticles as carriers for controlled herbicide diquat dibromide release through electrostatic interaction, Int. J. Mol. Sci. 20 (2019) 1330. <https://doi.org/10.3390/ijms20061330>.

[78] S. Sharma, S. Singh, A.K. Ganguli, V. Shanmugam, Anti-drift nano-stickers made of graphene oxide for targeted pesticide delivery and crop pest control, Carbon 115 (2017) 781-790. <https://doi.org/10.1016/j.carbon.2017.01.075>.

[79] W. Liang, J. Cheng, J. Zhang, Q. Xiong, M. Jin, J. Zhao, pH-responsive on-demand alkaloids release from core-shell ZnO@ZIF-8 nanosphere for synergistic control of bacterial wilt disease, ACS Nano 16 (2022) 2762-2773. <https://doi.org/10.1021/acsnano.1c09724>.

[80] C. Xu, L. Cao, P. Zhao, Z. Zhou, C. Cao, F. Li, Q. Huang, Emulsion-based synchronous pesticide encapsulation and surface modification of mesoporous silica nanoparticles with carboxymethyl chitosan for controlled azoxystrobin release, Chem. Eng. J. 348 (2018) 244-254. <https://doi.org/10.1016/j.cej.2018.05.008>.

[81] C. Xu, Y. Shan, M. Bilal, B. Xu, L. Cao, Q. Huang, Copper ions chelated mesoporous silica nanoparticles via dopamine chemistry for controlled pesticide release regulated by coordination bonding, Chem. Eng. J. 395 (2020) 125093. <https://doi.org/10.1016/j.cej.2020.125093>.

[82] S. Sharma, B. Singh, P. Bindra, P. Panneerselvam, N. Dwivedi, A. Senapati, A. Adholeya, V. Shanmugam, Triple-smart eco-friendly chili anthracnose control agro-nanocarrier, ACS Appl. Mater. Interfaces 13 (2021) 9143-9155. <https://doi.org/10.1021/acsami.0c18797>.

[83] Y. Huang, Y. Yang, B. Liang, S. Lu, X. Yuan, Z. Jia, J. Liu, Y. Liu, Green nanopesticide: pH-responsive eco-friendly pillar[5]arene-modified selenium nanoparticles for smart delivery of carbendazim to suppress sclerotinia diseases, ACS Appl. Mater. Interfaces 15 (2023) 16448-16459. <https://doi.org/10.1021/acsami.2c23241>.

[84] A.E. Kaziem, L. Yang, Y. Lin, A.E. Kazem, H. Xu, Z. Zhang, Pathogenic invasion-responsive carrier based on mesoporous silica/β-glucan nanoparticles for smart delivery of fungicides, ACS Sustainable Chem. Eng. 9 (2021) 9126-9138. <https://doi.org/10.1021/acssuschemeng.1c02962>.

[85] L. Yang, H. Chen, W. Yan, S. Huang, D. Cheng, H. Xu, Z. Zhang, A pH- and redox-stimulated responsive hollow mesoporous silica for triggered delivery of fungicides to control downy mildew of *Luffa cylindrica*, Pest Manage. Sci. 78 (2022) 3365-3375. <https://doi.org/10.1002/ps.6964>.

[86] Y. Tong, L. Shao, X. Li, J. Lu, H. Sun, S. Xiang, Z. Zhang, Y. Wu, X. Wu, Adhesive and stimulus-responsive polydopamine-coated graphene oxide system for pesticide-loss control, J. Agric. Food Chem. 66 (2018) 2616-2622. <https://doi.org/10.1021/acs.jafc.7b05500>.

[87] Y. Liang, C. Fan, H. Dong, W. Zhang, G. Tang, J. Yang, N. Jiang, Y. Cao, Preparation of MSNs-chitosan@prochloraz nanoparticles for reducing toxicity and improving release properties of prochloraz, ACS Sustainable Chem. Eng. 6 (2018) 10211-10220. <https://doi.org/10.1021/acssuschemeng.8b01511>.

[88] T.M. Abdelrahman, X. Qin, D. Li, I.A. Senosy, M. Mmby, H. Wan, J. Li, S. He, Pectinase-responsive carriers based on mesoporous silica nanoparticles for improving the translocation and fungicidal activity of prochloraz in rice plants, Chem. Eng. J. 404 (2021) 126440. <https://doi.org/10.1016/j.cej.2020.126440>.

[89] L.T. Wu, H. Pan, W.L. Huang, M.J. Wang, Z.X. Hu, F. Zhang, Self-assembled degradable iron-doped mesoporous silica nanoparticles for the smart delivery of prochloraz to improve plant protection and reduce environmental impact, Environ. Technol. Innovation 28 (2022) 102890. <https://doi.org/10.1016/j.eti.2022.102890>.

[90] Y. Gao, Y. Liu, X. Qin, Z. Guo, D. Li, C. Li, H. Wan, F. Zhu, J. Li, Z. Zhang, S. He, Dual stimuli-responsive fungicide carrier based on hollow mesoporous silica/hydroxypropyl cellulose hybrid nanoparticles, J. Hazard. Mater. 414 (2021) 125513. <https://doi.org/10.1016/j.jhazmat.2021.125513>.

[91] J. Dong, X. Liu, Y. Chen, W. Yang, X. Du, User-safe and efficient chitosan-gated porous carbon nanopesticides and nanoherbicides, J. Colloid Interface Sci. 594 (2021) 20-34. <https://doi.org/10.1016/j.jcis.2021.03.001>.

[92] J. Dong, W. Chen, D. Qin, Y. Chen, J. Li, C. Wang, Y. Yu, J. Feng, X. Du, Cyclodextrin polymer-valved MoS_2_-embedded mesoporous silica nanopesticides toward hierarchical targets via multidimensional stimuli of biological and natural environments, J. Hazard. Mater. 419 (2021) 126404. <https://doi.org/10.1016/j.jhazmat.2021.126404>.

[93] K. Wei, K. Zhao, Y. Gao, H. Zhang, X. Yu, M.-H. Li, J. Hu, Near-infrared-light responsive degradable polysulfide pesticide carriers by one-pot inverse vulcanization, Chem. Eng. J. 462 (2023) <https://doi.org/10.1016/j.cej.2023.142191>.

[94] J. Liu, X. Zhu, X. Chen, Y. Liu, Y. Gong, G. Yuan, J. Liu, L. Chen, Defense and inhibition integrated mesoporous nanoselenium delivery system against tomato gray mold, Environ. Sci.: Nano 7 (2020) 210-227. <https://doi.org/10.1039/C9EN00859D>.

[95] Y. Ji, S. Ma, S. Lv, Y. Wang, S. Lu, M. Liu, Nanomaterials for targeted delivery of agrochemicals by an all-in-one combination strategy and deep learning, ACS Appl. Mater. Interfaces 13 (2021) 43374-43386. <https://doi.org/10.1021/acsami.1c11914>.

[96] Y. Chi, G. Zhang, Y. Xiang, D. Cai, Z. Wu, Fabrication of a temperature-controlled-release herbicide using a nanocomposite, ACS Sustainable Chem. Eng. 5 (2017) 4969-4975. <https://doi.org/10.1021/acssuschemeng.7b00348>.

[97] Y.B. Xiang, G.L. Zhang, Y. Chi, D.Q. Cai, Z.Y. Wu, Fabrication of a controllable nanopesticide system with magnetic collectability, Chem. Eng. J. 328 (2017) 320-330. <https://doi.org/10.1016/j.cej.2017.07.046>.

[98] C. Chen, G. Zhang, Z. Dai, Y. Xiang, B. Liu, P. Bian, K. Zheng, Z. Wu, D. Cai, Fabrication of light-responsively controlled-release herbicide using a nanocomposite, Chem. Eng. J. 349 (2018) 101-110. <https://doi.org/10.1016/j.cej.2018.05.079>.

[99] Y. Gao, Y. Zhang, S. He, Y. Xiao, X. Qin, Y. Zhang, D. Li, H. Ma, H. You, J. Li, Fabrication of a hollow mesoporous silica hybrid to improve the targeting of a pesticide, Chem. Eng. J. 364 (2019) 361-369. <https://doi.org/10.1016/j.cej.2019.01.105>.

[100] Y. Ding, Z. Xiao, F. Chen, L. Yue, C. Wang, N. Fan, H. Ji, Z. Wang, A mesoporous silica nanocarrier pesticide delivery system for loading acetamiprid: Effectively manage aphids and reduce plant pesticide residue, Sci. Total Environ. 863 (2023) 160900. <https://doi.org/10.1016/j.scitotenv.2022.160900>.

[101] A.E. Kaziem, Y. Gao, Y. Zhang, X. Qin, Y. Xiao, Y. Zhang, H. You, J. Li, S. He, α-Amylase triggered carriers based on cyclodextrin anchored hollow mesoporous silica for enhancing insecticidal activity of avermectin against *Plutella xylostella*, J. Hazard. Mater. 359 (2018) 213-221. <https://doi.org/10.1016/j.jhazmat.2018.07.059>.

[102] Y. Liang, Y. Gao, W. Wang, H. Dong, R. Tang, J. Yang, J. Niu, Z. Zhou, N. Jiang, Y. Cao, Fabrication of smart stimuli-responsive mesoporous organosilica nano-vehicles for targeted pesticide delivery, J. Hazard. Mater. 389 (2020) 122075. <https://doi.org/10.1016/j.jhazmat.2020.122075>.

[103] L. Hao, L. Gong, L. Chen, M. Guan, H. Zhou, S. Qiu, H. Wen, H. Chen, X. Zhou, M. Akbulut, Composite pesticide nanocarriers involving functionalized boron nitride nanoplatelets for pH-responsive release and enhanced UV stability, Chem. Eng. J. 396 (2020) 125233. <https://doi.org/10.1016/j.cej.2020.125233>.

[104] J. Yang, J. Feng, K. He, Z. Chen, W. Chen, H. Cao, S. Yuan, Preparation of thermosensitive buprofezin‐loaded mesoporous silica nanoparticles by the sol–gel method and their application in pest control, Pest Manage. Sci. 77 (2021) 4627-4637. <https://doi.org/10.1002/ps.6502>.

[105] Y. Gao, Y. Liang, Z. Zhou, J. Yang, Y. Tian, J. Niu, G. Tang, J. Tang, X. Chen, Y. Li, Y. Cao, Metal-organic framework nanohybrid carrier for precise pesticide delivery and pest management, Chem. Eng. J. 422 (2021) 130143. <https://doi.org/10.1016/j.cej.2021.130143>.

[106] A.E. Kaziem, Y. Gao, S. He, J. Li, Synthesis and insecticidal activity of enzyme-triggered functionalized hollow mesoporous silica for controlled release, J. Agric. Food Chem. 65 (2017) 7854-7864. <https://doi.org/10.1021/acs.jafc.7b02560>.

[107] Y. Xiang, G. Zhang, C. Chen, B. Liu, D. Cai, Z. Wu, Fabrication of a pH-responsively controlled-release pesticide using an attapulgite-based hydrogel, ACS Sustainable Chem. Eng. 6 (2018) 1192-1201. <https://doi.org/10.1021/acssuschemeng.7b03469>.

[108] G. Teng, C. Chen, N. Jing, C. Chen, Y. Duan, L. Zhang, Z. Wu, J. Zhang, Halloysite nanotubes-based composite material with acid/alkali dual pH response and foliar adhesion for smart delivery of hydrophobic pesticide, Chem. Eng. J. 451 (2023) 139052. <https://doi.org/10.1016/j.cej.2022.139052>.

[109] M. Wan, S. Song, X. Jiang, Z. Liu, Y. Luo, X. Gao, J. Liu, J. Shen, Tannic acid-modified MXene as a nanocarrier for the delivery of β-cyfluthrin as a sustained release insecticide, ACS Appl. Nano Mater. 5 (2022) 15583-15591. <https://doi.org/10.1021/acsanm.2c03630>.

[110] K. Chen, G. Yu, F. He, Q. Zhou, D. Xiao, J. Li, Y. Feng, A pH-responsive emulsion stabilized by alginate-grafted anisotropic silica and its application in the controlled release of λ-cyhalothrin, Carbohydr. Polym. 176 (2017) 203-213. <https://doi.org/10.1016/j.carbpol.2017.07.046>.

[111] Y. Wang, S. Song, X. Chu, W. Feng, J. Li, X. Huang, N. Zhou, J. Shen, A new temperature-responsive controlled-release pesticide formulation – poly(*N*-isopropylacrylamide) modified graphene oxide as the nanocarrier for lambda-cyhalothrin delivery and their application in pesticide transportation, Colloids Surf., A 612 (2021) 125987. <https://doi.org/10.1016/j.colsurfa.2020.125987>.

[112] S. Song, M. Wan, Y. Luo, H. Shen, J. Shen, Carboxymethyl chitosan-modified graphene oxide as a multifunctional vector for deltamethrin delivery and pH-responsive controlled release, enhanced leaf affinity, and improved mosquito-killing activity, Langmuir 38 (2022) 12148-12156. <https://doi.org/10.1021/acs.langmuir.2c01669>.

[113] W. Fu, K. Du, Z. Xu, J. Cheng, Z. Li, X. Shao, Dual photo-controlled release system for fipronil and dinotefuran, Photochem. Photobiol. Sci. 22 (2023) 825-836. <https://doi.org/10.1007/s43630-022-00355-4>.

[114] S. Song, Y. Wang, J. Xie, B. Sun, N. Zhou, H. Shen, J. Shen, Carboxymethyl chitosan modified carbon nanoparticle for controlled emamectin benzoate delivery: Improved solubility, pH-responsive release, and sustainable pest control, ACS Appl. Mater. Interfaces 11 (2019) 34258-34267. <https://doi.org/10.1021/acsami.9b12564>.

[115] W. Wu, M. Wan, Q. Fei, Y. Tian, S. Song, H. Shen, J. Shen, PDA@Ti_3_C_2_T*_x_* as a novel carrier for pesticide delivery and its application in plant protection: NIR-responsive controlled release and sustained antipest activity, Pest Manage. Sci. 77 (2021) 4960-4970. <https://doi.org/10.1002/ps.6538>.

[116] B. Liu, J. Zhang, C. Chen, D. Wang, G. Tian, G. Zhang, D. Cai, Z. Wu, Infrared-light-responsive controlled-release pesticide using hollow carbon microspheres@polyethylene glycol/α-cyclodextrin gel, J. Agric. Food Chem. 69 (2021) 6981-6988. <https://doi.org/10.1021/acs.jafc.1c01265>.

[117] Y. Gao, Y. Xiao, K. Mao, X. Qin, Y. Zhang, D. Li, Y. Zhang, J. Li, H. Wan, S. He, Thermoresponsive polymer-encapsulated hollow mesoporous silica nanoparticles and their application in insecticide delivery, Chem. Eng. J. 383 (2020) 123169. <https://doi.org/10.1016/j.cej.2019.123169>.
